# Inflammation and Vascular Effects after Repeated Intratracheal Instillations of Carbon Black and Lipopolysaccharide

**DOI:** 10.1371/journal.pone.0160731

**Published:** 2016-08-29

**Authors:** Daniel Vest Christophersen, Nicklas Raun Jacobsen, Ditte Marie Jensen, Ali Kermanizadeh, Majid Sheykhzade, Steffen Loft, Ulla Vogel, Håkan Wallin, Peter Møller

**Affiliations:** 1 Department of Public Health, Section of Environmental Health, University of Copenhagen, Copenhagen K, Denmark; 2 The National Research Centre for the Working Environment, Copenhagen, Denmark; 3 Department of Drug Design and Pharmacology, Section of Molecular and Cellular Pharmacology, Faculty of Health Sciences, University of Copenhagen, Copenhagen, Denmark; 4 Department of Micro- and Nanotechnology, Technical University of Denmark, Kgs. Lyngby, Denmark; GERMANY

## Abstract

Inflammation and oxidative stress are considered the main drivers of vasomotor dysfunction and progression of atherosclerosis after inhalation of particulate matter. In addition, new studies have shown that particle exposure can induce the level of bioactive mediators in serum, driving vascular- and systemic toxicity. We aimed to investigate if pulmonary inflammation would accelerate nanoparticle-induced atherosclerotic plaque progression in *Apolipoprotein E* knockout (*ApoE*^*-/-*^) mice. *ApoE* ^*-/-*^ mice were exposed to vehicle, 8.53 or 25.6 μg nanosized carbon black (CB) alone or spiked with LPS (0.2 μg/mouse/exposure; once a week for 10 weeks). Inflammation was determined by counting cells in bronchoalveolar lavage fluid. *Serum Amyloid A3* (*Saa3*) expression and glutathione status were determined in lung tissue. Plaque progression was assessed in the aorta and the brachiocephalic artery. The effect of vasoactive mediators in plasma of exposed *ApoE*^*-/-*^ mice was assessed in aorta rings isolated from naïve C57BL/6 mice. Pulmonary exposure to CB and/or LPS resulted in pulmonary inflammation with a robust influx of neutrophils. The CB exposure did not promote plaque progression in aorta or BCA. Incubation with 0.5% plasma extracted from CB-exposed *ApoE*^*-/-*^ mice caused vasoconstriction in aorta rings isolated from naïve mice; this effect was abolished by the treatment with the serotonin receptor antagonist Ketanserin. In conclusion, repeated pulmonary exposure to nanosized CB and LPS caused lung inflammation without progression of atherosclerosis in *ApoE*^*-/-*^ mice. Nevertheless, plasma extracted from mice exposed to nanosized CB induced vasoconstriction in aortas of naïve wild-type mice, an effect possibly related to increased plasma serotonin.

## Introduction

Cardiovascular disease is the leading cause of mortality worldwide, with exposure to inhaled airborne particulate matter being a major contributor to the burden of disease [1]. Exposure to particulate matter from ambient air or nanomaterials (NMs) is associated with vascular dysfunction and progression of atherosclerosis, which is believed to be promoted by inflammation, acute phase response and oxidative stress in the lungs and target tissue [2–5]. The role of pulmonary inflammation in particle-induced cardiovascular outcomes has been debated with experimental evidence ranging from the spill-over effect of cytokines from the lung to the circulation [6] to vascular effects occurring independently of pulmonary inflammation [7].

A number of studies have addressed cardiovascular effects of carbon black (CB) exposure in animal models. CB is a widely used carbon-based material in both bulk form and as an NM. The latter has a high particle number to mass ratio and unique surface properties, which make them attractive component in many products, although the small size and large surface area potentially make them more toxic than their larger counterparts [8]. It has been shown that pulmonary exposure to CB by intratracheal instillation (i.t.) once a week for ten weeks in LDL receptor knockout mice on a cholesterol-rich diet promoted progression of atherosclerotic plaque in the aorta [9]. There has also been reported a decrease in the vasorelaxation response in aortic segments from *ApoE*^*-/-*^ mice after two i.t. instillations of nanosized CB, whereas the same dose did not cause plaque progression in the aorta or the brachiocephalic artery (BCA) [10]. Nevertheless, 4 weeks inhalation by nose-only exposure to CB did not affect the vasoconstriction and vasorelaxation response in the aorta of rats [11]. Particle-generated vasomotor dysfunction may be related to relatively acute exposures because it has been shown that exposure to nanosized CB did not alter the vasorelaxation response in second order branch interlobar pulmonary arteries of male Wistar rats at day 21 after an i.t. instillation [12]. The same pattern was observed in dyslipidemic Zucker rats following oral administration of CB for 10 weeks; vasomotor dysfunction in the aorta was observed 24 h after the last exposure, whereas this particle-effect had subsided at 13 weeks post-exposure [13]. The mechanistic link between pulmonary/intestinal exposure to CB and cardiovascular outcomes has not been elucidated. The direct application of nanosized CB to artery segments caused dysfunction of the vasorelaxation response and increased vasoconstriction[14, 15]. However, these *ex vivo* observations are based on concentrations that are much higher than the concentrations that may be relevant in physiological scenarios after pulmonary exposure. The translocation of poorly soluble particles is typically much less than 1% of the deposited dose in the lungs, whereas higher translocation can ben observed for nanomaterials with high solubility such as zincoxide or nanosilver [16]. In addition, systemic low-grade inflammation has been hypothetized to be a mechanism of vascular effects following pulmonary exposure to particulate matter, but a recent systematic review and meta-analysis of the literature do not demonstrate any consistency between systemic inflammation and vascular endpoints [17]. Interestingly, recent studies have shown that pulmonary exposure to particles or ozone can induce the level of bioactive mediators in serum that acts as drivers for systemic toxicity, including loss of vascular integrity and vasomotor dysfunction [18–20]. As such, it is difficult to disentangle the contribution of pulmonary inflammation from other mediators on vascular effects in CB exposed animals because the material dose-dependently causes a strong pulmonary inflammation response [21].

The aim of this study was to investigate if pulmonary inflammation exacerbated nanosized CB induced progression of atherosclerosis. LPS was used as a non-particulate agent because it induces inflammation and accelerates plaque progression in *ApoE*^*-/-*^ mice [22]. We carried out a range-finding experiment to find a dose of LPS that would produce a similar extent of pulmonary inflammation as the CB material of choice (i.e. Printex 90). Subsequently, we assessed pulmonary inflammation and atherosclerotic plaque progression after CB and/or LPS exposure in *ApoE*^*-/-*^ mice. Plaque progression was evaluated in the aorta and BCA; both arteries are prone to develop atherosclerotic lesions in *ApoE*^*-/-*^ mice. Pulmonary inflammation was assessed as a neutrophilic influx in bronchoalveolar lavage fluid (BALF) and the acute phase response as gene expression of *Serum Amyloid A3* (*Saa3*) in lung tissue. The level of oxidative stress in the lungs was assessed by measuring the glutathione balance. Finally, we investigated vasoactive effects of mediators in plasma from nanosized CB, and/or LPS exposed *ApoE*^*-/-*^ mice in aortas of naïve wild-type C57BL/6 mice (the *ApoE*^*-/-*^ background strain).

## Material and Methods

### Particles

CB was chosen as a high volume industrial NM. Specifically, we used Printex 90 because it is well-characterized physicochemically and it has been used in several studies as benchmark type on NM that causes both pulmonary inflammation and oxidative stress [23–30]. Printex 90 was a kind gift from Degussa-Hüls, Frankfurt, Germany. We have previously analyzed and reported the physicochemical characteristics of the Printex 90 material [31].

Printex 90 was suspended in nanopure water (< 45 μm pore size) to a concentration of 2.5 mg/ml and sonicated in an ice bath for 16 min with 10% amplitude using a Branson Sonifier S-450D (Branson Ultrasonics Corp., Danbury, CT, USA) equipped with a disruptor horn (Model number: 101-147-037). The instillation vehicle was prepared as described above without particles. For LPS spiked suspensions, 4 μg/ml LPS (*Escherichia coli* 055: B5, Sigma-Aldrich) was added to the sonicated suspension with or without particles and vortexed for 2 min before i.t. instillation. A fresh suspension was prepared immediately before i.t. instillation.

Dynamic light scattering (DLS) Zetasizer Nano ZS (Malvern Instruments Ltd., UK) was used to measure the hydrodynamic size distribution of CB in nanopure water as described previously [29]. Calculations were done by the DTS software using the viscosity of water. S1 Fig depicts particle size in the suspensions. In brief, the particle size of CB was 44 and 38 nm in the low and high dose groups, respectively. Addition of LPS increased the particle size of the suspension to 1281 and 1718 nm, respectively. Likewise, the polydispersity index of the suspensions with CB were lower as compared to the suspensions with CB and LPS.

### Animal and caging conditions

For the LPS pilot study, female C57BL/6-Ntac mice were purchased from Taconic (Ejby, Denmark) at nine weeks of age and allowed one week of acclimatization before the first exposure. For the main studies, female C57BL/6-Apoe^tm1^ *ApoE*^*-/-*^ mice were purchased from Taconic (Ejby, Denmark) at eight weeks of age and allowed two weeks of acclimation before the experimental procedure. All mice were randomly assigned to groups of 10 animals and housed in polypropylene cages (Jeluxyl HW 300/500) with sawdust bedding and enrichment, such as pinewood sticks and rodent tunnels. The mice were maintained on a 12:12 h light-dark cycle and with controlled humidity and temperature. Mice had ad libitum access to regular mouse chow (Altromin no. 1324 Christian Petersen, Denmark) and tap water. All animal procedures followed the local institutional and governmental guidelines on animal ethics and welfare issued by the Danish government and the Animal Experimental Inspectorate under the Ministry of Justice approved the study (permission 2010/561-1779).

### Study design

#### LPS pilot study

The LPS dose-range study was designed to find a dose of LPS that in C57BL/6 mice when repeatedly administered, would give a neutrophil influx in BALF similar to that of CB. Furthermore, the purpose of the experiment was to select doses of CB and LPS that would not cause a saturation of the inflammatory response. In the assessment of dose-response relationship, C57BL/6 mice were exposed by i.t. instillation to vehicle, 0.047 μg, 0.094 μg, 0.187 μg, 0.375 μg, 0.750 μg, 1.5 μg, 3.0 μg or 6.0 μg of LPS and euthanized at 24 h post-exposure (n = 3 mice/group). This was followed by a repeated exposure study in which C57BL/6 mice were exposed by i.t. instillation to vehicle, 0.2 μg (low-dose) or 1.0 μg (high-dose) of LPS once a week for four weeks (n = 3 mice/group). The total dose in the repeated exposure study was 0.8 μg (low-dose) and 4 μg (high-dose). The mice were euthanized at 24 h after the last exposure, and the BALF was collected. The vehicle utilized in these experiments was 2% serum obtained from sibling mice diluted with nanopure water under sterile conditions.

#### Main study 1

This study was conducted as a 2x3 factorial design with six exposure groups (n = 10/group) of *ApoE*^*-/-*^ mice as follows: 1) vehicle (nanopure water), 2) vehicle spiked with LPS, 3) low-dose CB, 4) low-dose CB spiked with LPS, 5) high-dose CB, and 6) high-dose CB spiked with LPS. All mice were exposed by i.t. instillation once a week for 10 weeks. The total dose given in the 10 administrations was 85.3 μg/mouse for the low-dose and 256 μg/mouse for high-dose nanosized CB and 2 μg/mouse for LPS spiked groups.

#### Main study 2

To increase the statistical power of plaque progression results for the second and third exposure groups, a second study was performed. In this study only the vehicle (nanopure water), vehicle spiked with LPS, and low-dose of CB groups were included. Ten *ApoE*^*-/-*^ mice in each group were exposed to vehicle, CB (total dose = 85.3 μg/mouse) or LPS (total dose = 2 μg/mouse) once a week for ten weeks by the same procedure as described in study 1.

The exposure doses of 8.53 and 25.6 μg/mouse of CB were selected from earlier observations on i.t. instillations in C57BL/6 mice that showed neutrophilic influx in BALF at the high dose [21, 25, 29, 32, 33]. S2 Fig depicts the dose-response relationship for total cells and neutrophils in earlier studies. Four repeated i.t. instillations of CB to pregnant C57BL/6 mice were not associated with increased influx of neutrophils at a weekly dose of 14 μg/mouse (total dose = 54 μg/mouse) [34]. Moreover, earlier observations in which CB exposure resulted in a stronger influx of neutrophils in BALF in *ApoE*^*-/-*^ mice as compared to wild-type counterparts were taken into account [21]. Based on these observations, it was anticipated that CB exposure would yield approximately 50.000 neutrophils in BALF. Hence, an LPS dose of 0.2 μg/mouse (total dose = 2 μg/mouse) was selected for the repeated exposure study in C57BL/6 mice. The lowest of exposure to CB (8.53 μg/mouse, corresponding to approximately 0.30 mg/kg bodyweight per week) is approximately equal to the accumulated dose that humans can encounter during a one-week stay in a working environment with the same aerosol concentration of respirable CB as the Danish threshold limit (i.e. aerosol concentration of 3.5 μg/m^3^, inhalation of 8 m^3^/day, 15% deposition in lower respiratory tract and 70 kg bodyweight).

### Intratracheal instillation

All mice were anesthetized using 3–4% isoflurane until fully relaxed and thereafter placed at a 50° angle on a board for the i.t. instillation. A diode light source was positioned on the larynx to visualize the trachea and vocal cords. The tongue was gently pressed towards the lower jaw, and the trachea was intubated using a 24 gauge BD Insyte plastic catheter (#381212, Becton Dickinson, Denmark) with a shortened needle. To ensure that the tube was positioned correctly in the trachea an extremely sensitive pressure transducer was used to measure the respiration frequency as previously described [21]. Each mouse was instilled with 50 μl of suspension, immediately followed by 200 μl of air using a 250 μl SGE glass syringe (250F-LT-GT, Micro- Lab, Aarhus, Denmark).

### Bronchoalveolar lavage and isolation of organs

At 24 h post-exposure, the mice were euthanized using a cocktail of Zoletil® (Tiletamine/Zolazepam), Fentanyl, and Rompun®. Their weight was recorded and blood collected by cardiac puncture in an eppendorf tube containing 36 μl K_2_EDTA. The plasma was collected by centrifugation. Immediately after blood collection, a lung lavage was performed by cannulation of the trachea using a 22 gauge needle equipped with polyurethane catheter. The lungs were flushed twice using sterile saline (2 x 0.8 ml/mouse). The total BALF volume recovery was estimated to be around 75%. The BALF was kept on ice until it was centrifuged (400 x g, 4°C for 10 min) and the supernatant stored at -80° until use.

The pellet was resuspended in 100 μl of media (HAMS F12 (GIBCO #21765) with 10% fetal bovine serum (FBS)). The total BALF cell count of each mouse was determined using 20 μl of cell suspension in an NC-100 Nucleocounter (ChemoMetec A/S, DK). To prepare Cytospin slides 40 μl of cell suspension was centrifugated at 1000 rpm for 4 min.

The BALF cells deposited on objective slides were stained using May-Grunwald and Giemsa co-staining. Differential cell count was performed counting 200 cells per slide by a person blinded to the exposure groups.

After the BAL procedure, the lungs and liver tissue were dissected, snap frozen in liquid nitrogen and stored at -80°C.

The heart and the whole aorta (from the arch to the iliac bifurcation) were dissected and transferred to a petri dish containing ice-cold PBS. The aortic arch and BCA was gently trimmed of fat- and connective tissue under an Olympus SZX7 stereomicroscope. The BCA was embedded in Tissue-Tek® O.C.T™ Compound (Sakura Finetek, Værløse, DK), frozen on dry ice and stored at -80°C. The heart and aorta were kept in phosphate buffer saline (PBS) at 4°C for a few hours before they were trimmed free of fat- and connective tissue.

### Glutathione quantification

Total glutathione was measured by reducing oxidized glutathione dimers (GSSG) with the addition of 7 μl of 10 mM sodium dithionite to all samples and incubating at room temperature for 1 h. Reduced glutathione was quantified in lung tissue from *ApoE*^*-/-*^ mice using an *o*-phthalaldehyde probe that reacts with the reduced form of glutathione, generating a fluorescence signal. Briefly, approximately 100 mg of tissue from the right lung was homogenized on ice utilizing an IKA ULTRA TURRAX^®^ T25 (disperser S25N-10G) in a redox quenching lysis buffer containing 5% trichloroacetic acid. Homogenates were vortex briefly and processed according to a slightly modified version of the protocol adapted from Senft and colleagues [35]. Lysates were diluted 1:10 for analysis and reduced glutathione normalized to the tissue mass.

### RNA purification and gene expression

Saber and colleagues have shown a robust increase in the gene expression of the acute phase reactant *Saa3* in the lungs after i.t. instillation of nanosized CB [36]. In addition, Bourdon et al have shown that the expression of *Saa3* in lungs is much higher and prolonged as compared to the expression of other acute phase proteins after a single i.t. instillation of CB [23]. Based on their findings we investigated the lung expression of *Saa3* in the present study because it is the most differentially expressed acute phase protein after i.t. instillation of CB in mice.

Mouse lung RNA was purified in an RNAse free environment using Maxwell® 16 LEV simplyRNA Tissue Kit (Promega, Madison, WI, USA). In brief, 16–22 mg of lung tissue in homogenization solution containing thioglycerol and stainless steel beads was homogenized on a Tissuelyser II (Qiagen, Hilden, Germany) 30 times/second for 60 seconds and stored on ice for a few min. The homogenates were transferred to Maxwell® 16 LEV cartridges (MCE) and purified on AS2000 Maxwell® 16 Instruments (Promega, Madison, WI, USA) according to manufacturer’s instructions. The RNA concentration and purity was measured on a Nanodrop 2000 UV-Vis Spectrophotometer (Thermo Scientific). All RNA samples with A260/280 ratio between 2.0–2.15 were used for cDNA synthesis.

cDNA was synthesized from DNAse treated RNA using Taq-Man® Reverse transcriptase reagents (Applied Biosystems) according to manufacturer’s protocol.

*Saa3* gene expression was measured using real-time qPCR with *18S* as reference gene as described previously [37]. In brief, we quantified the relative expression levels of *Saa3* and *18S* genes using commercial Taq-Man 2xPCR master mix (Applied Biosystems) on a Viia7 sequence detector (Applied Biosystems). cDNA from each lung sample was run in triplicates with target and reference gene in separate wells. Negative controls (i.e. no RNA had been converted to cDNA), a sample without RNA and cDNA, and a plate control were included in each run, the latter to control for day to day variation. The sequences of the *Saa3* primers and probe were: *Saa3* forward: 59 GCC TGG GCT GCT AAA GTC AT 39, *Saa3* reverse: 59 TGC TCC ATG TCC CGT GAA C 39 and *Saa3* probe: 59 FAM–TCT GAA CAG CCT CTC TGG CAT CGC T–TAMRA 39. The relative expression of the target gene was quantified using the comparative method 2^-ΔCT^ [38].

### Atherosclerotic plaque progression in the aorta

The trimmed aorta was cut longitudinally from the arch to the iliac bifurcation, flattened and mounted between an objective glass and a cover slide, avoiding any overlapping tissue. Digital images of the intimal surface were obtained using an Olympus SZX7 stereomicroscope and Olympus Color View I camera. The level of atherosclerotic lesions (plaque percentage of the total surface area of the aorta) was quantified by a person blinded to the exposure groups using image processing software ImageJ.

### Atherosclerotic plaque progression in brachiocephalic arteries

Triplicates of 5 μm thick frozen sections were obtained at 100 and 200 μm distal to the aortic arch on a CM3050 S-Cryostat (Leica Microsystems Nussloch GmbH, DE). The sections were mounted on Super Frost® Plus slides (Thermo Scientific) and stored at -80°C. The mounted sections were fixed in Bouin’s solution (Sigma) overnight, followed by staining with Masson's trichrome stain (Sigma). In brief, sections were stained in Weigert’s Iron Hematoxylin Solution (Ampliqon, DK) for 2 min; Biebrich Scarlet-Acid Fuchsin (HT15-1, Sigma) for 5 min; Phosphotungstic Acid/Phosphomolybdic Acid workings solution (HT152/HT153, Sigma) for 5 min, and Aniline Blue for 5 min. The sections were destained in 1% acetic acid for 2 min, dehydrated through graded ethanol to xylene, and mounted with Pertex for examination by light microscopy. Digital images were captured using an Olympus BX41 microscope and Olympus Color View I camera or using the Hamamatsu NDP slide scanner (Hamamatsu Nanozoomer 2.0HT). Image analysis was carried out using ImageJ or by analyzing the virtual slides by using the Hamamatsu NDP viewer. All analyses were performed by a person blinded to the exposure groups. Each data point was calculated as the mean of triplicates from 100 and 200 μm distal to the aortic arch, respectively (the combination of the mean of the triplicates from the 100 and 200 μm cross-sections was due to no difference being observed in the plaque area between the locations).

Plaque progression in the BCA was calculated as the percentage of plaques covering the lumen cross-sectional area. The relative plaque area was evaluated by calculating the intima-media ratio.

Classification of atherosclerotic plaques in the BCA was carried out using the American Heart Association guideline [39]. In brief, stages I-III is regarded as clinically silent lesions that are precursors to advanced lesions. Stage IV is advanced lesions called atheroma and has a core of accumulated extracellular lipid. Stage V lesions are advanced fibroatheroma lesions that have multiple lipid cores, fibrotic layers, and calcifications. Stage VI lesions are complicated lesion with surface rupture and hematoma-hemorrhage thrombus, these are not observed in *ApoE*^*-/-*^ mice and, therefore, the score is not used in this study [39]. S3 Fig depicts examples of the stages of lesions in the present study.

### Effects of vasomotor active mediators in plasma

The aorta of naïve female C57BL/6 Ntac mice (Taconic, Ejby, Denmark) aged 10–12 weeks was dissected and trimmed free of fat and connective tissue in oxygenated cold physiological saline solution (PSS) buffer after cervical dislocation, as described previously [40]. The thoracic section of the aorta was cut into ring-shaped segments of 2 mm. Two stainless steel wires, 40 μm in diameter, were gently led through the lumen of each ring segment and mounted in the organ bath of a Multi-Wire Myograph 620M (Danish Myo Technology, Aarhus, DK) interfaced to a PowerLab 4/35 recorder (ADInstruments). Each organ bath contained 5 ml of cold oxygenated PSS and was continuously perfused with a 95% O_2_ and 5% CO_2_ gas mixture. After mounting the segments, the heat was turned on, fresh 37°C warm PSS added and the segments were allowed to equilibrate. Next the passive length-tension relationships of each segment were determined using the DMT LabChart normalization procedure. After a successful normalization, the vessel viability was confirmed via non-receptor mediated contraction. In brief, 5 ml of K-PSS was added to the organ bath while the contraction was allowed to stabilize (reached a plateau). Thereafter, the vessels were stimulated three times with 37°C K-PSS. Only viable vessels were used and stimulated with plasma from *ApoE*^*-/-*^ mice that had been exposed to vehicle, LPS or low-dose CB in order to investigate the vasomotor response. The aorta ring segments were stimulated with 0.5% plasma, and the presence or absence of vessel contraction recorded. At this juncture, an additional 0.5% of plasma was added to the vessel and contraction evaluated. As a final phase, 10 μM of acetylcholine (Ach) was added to assess the endothelium-dependent vasorelaxation (endothelium function). This concentration of Ach induces maximal relaxation of prostaglandin F_2α_ pre-contracted aorta rings. In addition, we have previously shown that exposure to nanomaterials affects the maximal endothelium-dependent vasorelaxation rather than the EC_50_ value [10, 41–43].

To investigate the origin of the pharmacological modulators in the plasma we antagonized the serotonin receptor with ketanserin. This drug is a S_2_-serotoninergic antagonist, which also inhibits α_1_-adrenergic receptors [44]. 1) Aorta ring segments were stimulated with 1% plasma (from CB-exposed *ApoE*^*-/-*^ mice), at the plateau of contraction the serotonin receptor antagonist Ketanserin (0.1 μM) was added and the force was recorded. 2) Aorta ring segments were pre-incubated 17 min with Ketanserin (0.1 μM), and 1% plasma (plasma from CB-exposed *ApoE*^*-/-*^ mice) was added, and the force was recorded. In addition, the aorta ring segments were stimulated with Phenylephrine (10 μM) to demonstrate whether the artery had preserved the ability to receptor-mediated contraction via a receptor different from serotonin receptors. The serotonin investigations were reproduced in 3 different mice (n = 3).

For data analysis, the response was ranked based upon contraction above 0.5 mN in the aorta ring segments. This difference was used for practical reasons because certain plasma samples produced little vasoconstriction, although the basal tonus increased slightly over the incubation period, whereas other samples produced a swift vasoconstriction. An increase in tonus of 0.5 mN was used as threshold for vasoconstriction because this difference is larger than the variation observed in the basal tonus of aorta rings. The threshold for a significant vasorelaxation response to 10 μM Ach was calculated as a decrease of more than 50% from the contraction following incubation with 0.5% plasma. The results of study 1 and 2 have been pooled in the statistical analysis to increase the power of the qualitative assessment.

### Endotoxin assay

To assess whether the CB contained LPS or inhibited the LPS-mediated response, a Limulus amebocyte lysate (LAL) gel clot assay (N194-06, Lonza, Walkersville, MD) was utilized. In brief, a suspension of 5 ml (2.5 mg/ml) CB was sonicated in endotoxin-free water for 16 min and used at the same concentration as the i.t. instillations. LPS (*Escherichia coli* 055: B5, Sigma-Aldrich) was added to a final concentration of 4 μg/ml, equivalent to 10,000 Endotoxin Unit/ml. The CB working solutions (8.53 μg/ml and 25.6 μg/ml) were serially diluted (two-fold dilution) for analysis and a standard curve was generated according to the manufacturer's protocol. A solid gel clot formation was interpreted as LPS being present in the sample or as no interference from the CB in the sample. The lack of clot formation as was interpreted as no LPS being present in the sample or that CB inhibited the effect of LPS.

### Statistics

The results of the LPS pilot study were analyzed by regression analysis fitted to either a four-parameter sigmoid dose-response (single-dose i.t. instillation) or linear (repeated i.t. instilations) curve. The BALF data of study 1 were analyzed by two-factor ANOVA (interaction analysis) followed by a Fisher’s least statistical difference (LSD) post-hoc test. BALF data for eosinophils were log-transformed to achieve homogeneity of variance (assessed by Bartlett’s test). For lymphocytes, neutrophils and epithelial cells, log-transformation did not produce homogeneity of variance and parametric test on ranks was used with Fisher’s LSD post-hoc test (fold-differences and 95% confidence interval (CI) have been calculated from data on the nominal scale). The BALF data in study 2 were analyzed by one-way ANOVA with Tukey’s post-hoc test. In study 1, data on plaque progression in the aorta and BCA and the intima-media ratio were analyzed by two-factor ANOVA (interaction analysis) with Fisher’s LSD post-hoc test. The data on plaque progression in the aorta and BCA and the intima/media ratio of study 2 were log-transformed to achieve homogeneity of variance and analyzed using one-way ANOVA with Tukey’s post-hoc test. The data for plasma effects on the aorta were analyzed using χ^2^-test (data from study 1 and 2 were pooled within their respective groups). The data on glutathione in study 1 was analyzed by two-factor ANOVA (interaction analysis) with Fisher’s LSD post hoc test and the data of study 2 using one-way ANOVA with Tukey’s post-hoc test. Data on *Saa3* gene expression were log-transformation to achieve homogeneity of variance and parametric test on ranks was used with Fisher’s LSD post-hoc test. The statistical analyzes were carried out in STATA 13 program package (StataCorp LP, College Station, TX, USA), Statistica version 5.5 (StatSoft Inc., Tusla, OK, USA) and GraphPad Prism version 5.00 for Windows, (GraphPad Software, San Diego, CA USA,). All results are reported as the mean and standard error of the mean (SEM). Statistical significance was accepted at 5% level, and all P-values refer to post-hoc tests.

## Results

### Pulmonary inflammation after exposure to LPS in wild-type C57BL/6 mice

**Fig 1:**
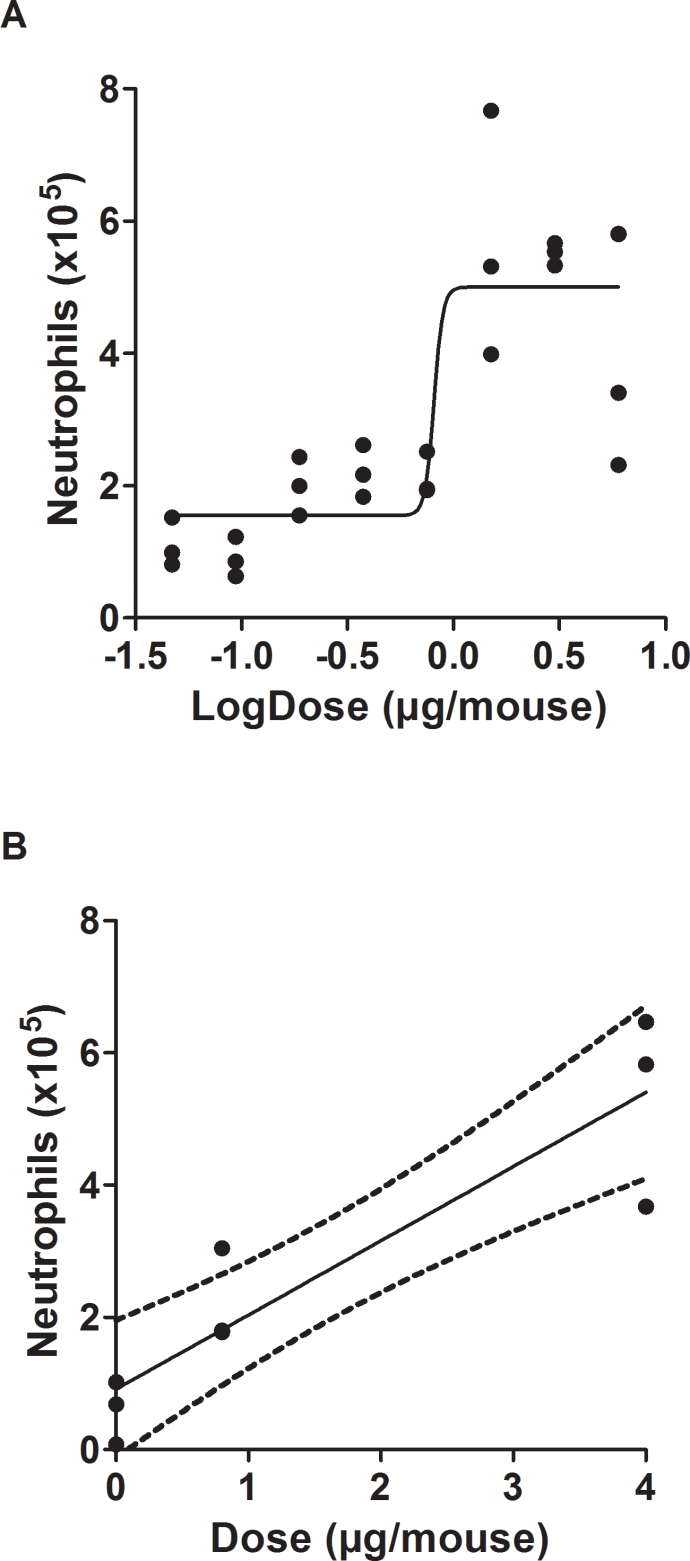
**The influx of neutrophils in BALF after a single (A) or repeated (B) i.t. instillation of lipopolysaccharide (LPS) in wild-type C57BL/6 mice**. Symbols represent the number of neutrophils in each mouse (mean values are reported in S1 Table). The regression lines represent sigmoid (A) and linear with 95% confidence interval (B) curve fit.

Pulmonary exposure to a single dose of LPS significantly increased the number of neutrophils in BALF (Fig 1). There were unaltered numbers of macrophages, eosinophils, lymphocytes or epithelial cells at any dose (S1 Table). The data fitted reasonably well to a sigmoid dose-response curve (R^2^ = 0.72), with the statistically significant bottom (1.6x10^5^ cells, 95% confidence interval: 0.9x10^5^ to 2.2x10^5^ cells) and top values (5.0x10^5^ cells, 95% confidence interval: 4.1x10^5^ to 5.9x10^5^ cells).

The repeated exposure to LPS during four weeks was associated with increased influx of neutrophils in BALF at the low (0.8 μg/mouse: 0.9x10^5^ cells, 95% confidence interval: 0.5x10^5^ to 1.3x10^5^ cells) and high dose (4.0 μg/mouse: 4.5x10^5^ cells, 95% confidence interval: 2.7x10^5^ to 6.3x10^5^ cells). The exposure did not significantly affect the numbers of other leukocytes or epithelial cells at any dose (S1 Table).

### Pulmonary inflammation in ApoE^-/-^ mice after repeated exposure to CB and/or LPS

#### Study 1

The statistical analysis showed an interaction between the exposure to CB and LPS for total cells (P<0.01), lymphocytes (P<0.01) and neutrophils (P<0.001). The exposure to LPS was associated with increased number of total cells (1.9x10^5^ cells, 95% CI: 0.9x10^5^ to 3.0x10^5^ cells), whereas there were slightly lower numbers of total cells in the LPS-exposed mice that also received high-dose CB (-0.5x10^5^ cells, 95% CI: -1.5x10^5^ to 0.6x10^5^ cells) relative to the LPS-exposed mice (Table 1). There was a strong and significant increase in the number of neutrophils in the low-dose CB (0.8x10^5^ cells, 95% CI: 0.1x10^5^ to 1.4x10^5^ cells) and LPS (2.5x10^5^ cells, 95% CI: 1.9x10^5^ to 3.1x10^5^ cells) exposed mice compared to the vehicle group. Additionally, there was a lower number of neutrophils in the low-dose CB+LPS (-1.0x10^5^ cells, 95% CI: -1.6x10^5^ to -0.3x10^5^ cells) and high-dose group CB+LPS (-0.6x10^5^ cells, 95% CI: -1.3x10^5^ to -0.02x10^5^ cells) groups as compared to the LPS group. The number of lymphocytes was increased in the low-dose (0.5x10^5^ cells, 95% CI: 0.3x10^5^ to 0.7x10^5^ cells) and high dose (0.2x10^5^ cells, 95% CI: 0.01x10^5^ to 0.3x10^5^ cells) CB exposed animals as compared to the controls. Moreover, the group of LPS exposed mice had decreased the number of epithelial cells as compared to the groups not exposed to LPS (P<0.01).

**Table 1:** BALF cell number and distribution at 24 h post-exposure after repeated i.t. instillations of carbon black (CB) and/or lipopolysaccharide (LPS) in *ApoE*^*-/-*^ mice.

Group	Vehicle	Low-dose CB	High-dose CB	LPS	Low-dose CB+LPS	High-dose CB+LPS
**Study 1**						
Total cells (x 10^3^)	187.2 ± 32.1	378.9 ± 39.7***	243.5 ± 37.7	405.0 ± 44.5*^##^	358.5 ± 39.0^##^	297.1 ± 30.2*^##^
Neutrophils (x 10^3^)	8.5 ± 5.2	85.2 ± 16.5***	52.1 ± 11.5***	261.0 ± 25.8^##^	164.5 ± 32.9*^##^^$^	196.5 ± 26.1*^##^^$^
Macrophage (x 10^3^)	98.1 ± 15.3	138.4 ± 10.2*	134.9 ± 13.4*	108.0 ± 13.2^##^	130.7 ± 14.7^##^	86.7 ± 12.9
Eosinophils (x 10^3^)	60.1 ± 20.7	87.2 ± 24.1	26.3 ± 15.7	20.0 ± 8.9	32.7 ± 7.8	5.4 ± 3.0^##^
Lymphocytes (x 10^3^)	3.2 ± 1.1	53.5 ± 9.3***	20.7 ± 7.3***	10.0 ± 2.4	20.3 ± 7.2	4.0 ± 0.9
Epithelial (x 10^3^)	17.1 ± 4.1	14.6 ± 3.8	9.4 ± 1.7	6.1 ± 2.7^##^	10.3 ± 2.1	4.5 ± 1.4^##^
**Study 2**						
Total cells (x 10^3^)	103.3 ± 17.4	104.8 ± 16.7	ND	294.4 ± 27.3***	ND	ND
Neutrophils (x 10^3^)	1.6 ± 0.6	11.6 ± 4.4***	ND	176.7 ± 23.3***	ND	ND
Macrophage (x 10^3^)	72.3 ± 12.1	66.2 ± 7.1	ND	98.1 ± 8.8	ND	ND
Eosinophils (x 10^3^)	14.7 ± 5.2	14.2 ± 7.9	ND	9.8 ± 3.3	ND	ND
Lymphocytes (x 10^3^)	1.8 ± 0.9	2.6 ± 0.8	ND	2.1 ± 0.8	ND	ND
Epithelial (x 10^3^)	13.0 ± 2.4	10.1 ± 2.1	ND	7.8 ± 2.1	ND	ND

* P<0.05

*** P<0.001 in CB exposed group compared to vehicle group.

^##^ P<0.01 in LPS group compared to vehicle control group.

^$^ P<0.05 in CB+LPS exposed group compared to LPS group.

Data is presented as mean ± SEM. The BALF cells were not determined (ND) in all groups in study 2.

#### Study 2

There was a significant increase in total cell number and neutrophil influx in BALF in the LPS exposed (P<0.0001) and low-dose CB exposed mice (P<0.001) (Table 1). There was no significant difference between the groups for macrophages, lymphocytes, eosinophils and epithelial cell.

### Increased expression of Saa3 in lung tissue of ApoE^-/-^ mice after pulmonary exposure to CB and/or LPS

**Fig 2:**
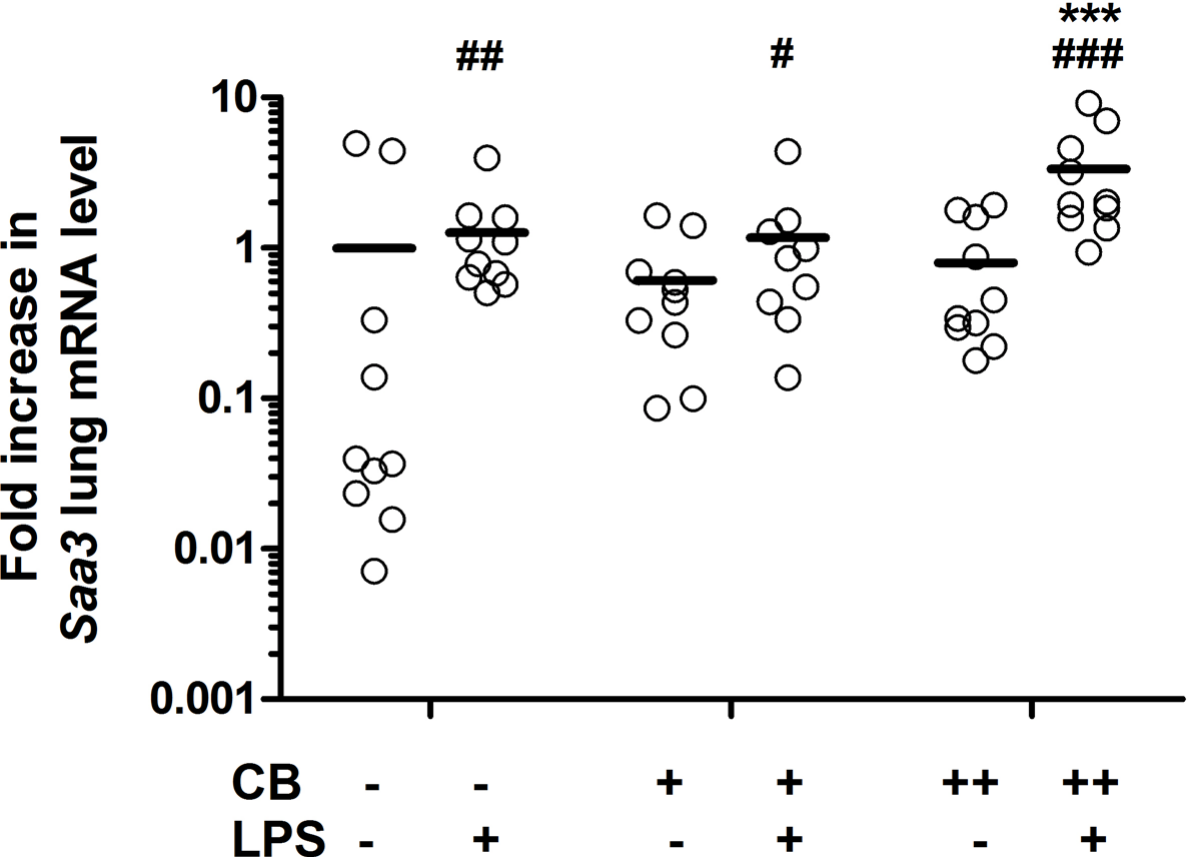
Normalized *Saa3* mRNA levels in lung tissue from *ApoE*^*-/-*^ mice after repeated i.t. instillations of carbon black (CB) and/or lipopolysaccharide (LPS). Fold increase in mRNA levels of *Saa3* compared to the vehicle group are shown. Open circles and squares represent the individual mice. Minus (-) denotes no exposure, plus denotes low (+) or high-dose (++) exposure. Lines represent the mean in each group (N = 9–10 per group). Asterisk denotes ***P<0.001 compared to the LPS exposed group, and ^#^P<0.05, ^##^P<0.01, ^###^P<0.001 compared to the vehicle control, two-factor ANOVA with Fisher’s LSD post-hoc test.

*Saa3* expression was measured as a marker of acute-phase response in lung tissue. It has previously been shown that nanoparticle-mediated neutrophil influx in BALF correlates with *Saa3* expression in mouse lung [5, 36]. In the present study, we only measured *Saa3* expression in study 1. The LPS exposure (P<0.01) and high-dose CB+LPS (P<0.001) caused a significant increase in the expression of *Saa3* in lung tissue 24 h post-exposure (Fig 2).

### Unaltered levels of glutathione status in lung tissue of ApoE^-/-^ mice after pulmonary exposure to CB and/or LPS

**Fig 3:**
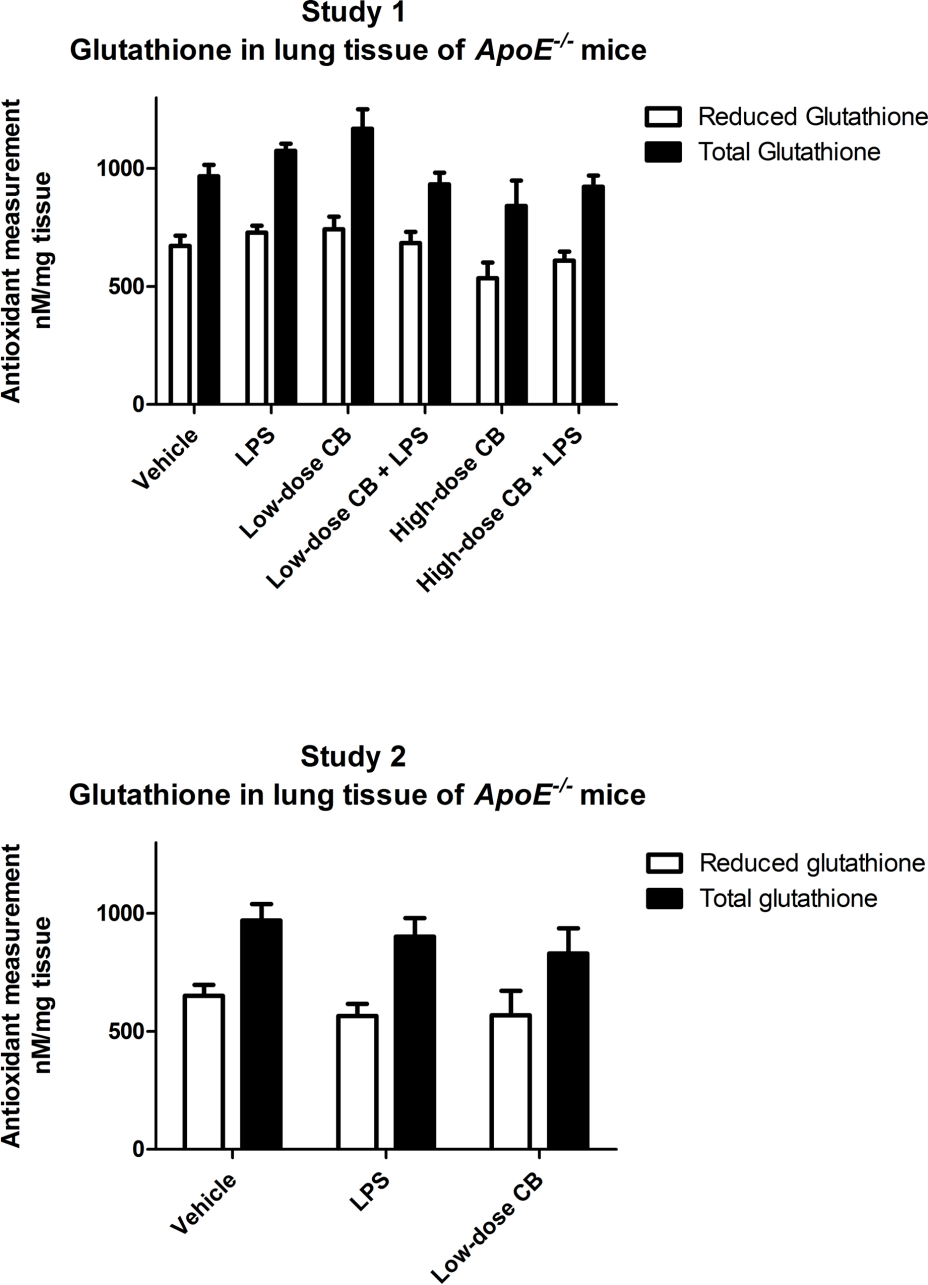
Glutathione status in lung tissue of *ApoE*^*-/-*^ mice after repeated i.t. instillations of carbon black (CB) or lipopolysaccharide (LPS). Total and reduced glutathione was measured at 24 h post-exposure in lung homogenate from *ApoE*^*-/-*^ mice of study 1 and 2. Data is presented as mean and SEM (n = 10 mice per group).

As a measure of oxidative stress, total and reduced glutathione were measured in lung homogenate 24 h after last exposure (Fig 3). There were no differences in total and reduced glutathione between the exposure groups and controls in both studies. In study 1 there was a trend of higher total glutathione levels in the low-dose CB exposure group, which was not observed in study 2.

### Unaltered progression of atherosclerosis in the aorta of ApoE^-/-^ mice after repeated exposure to CB and/or LPS

**Fig 4:**
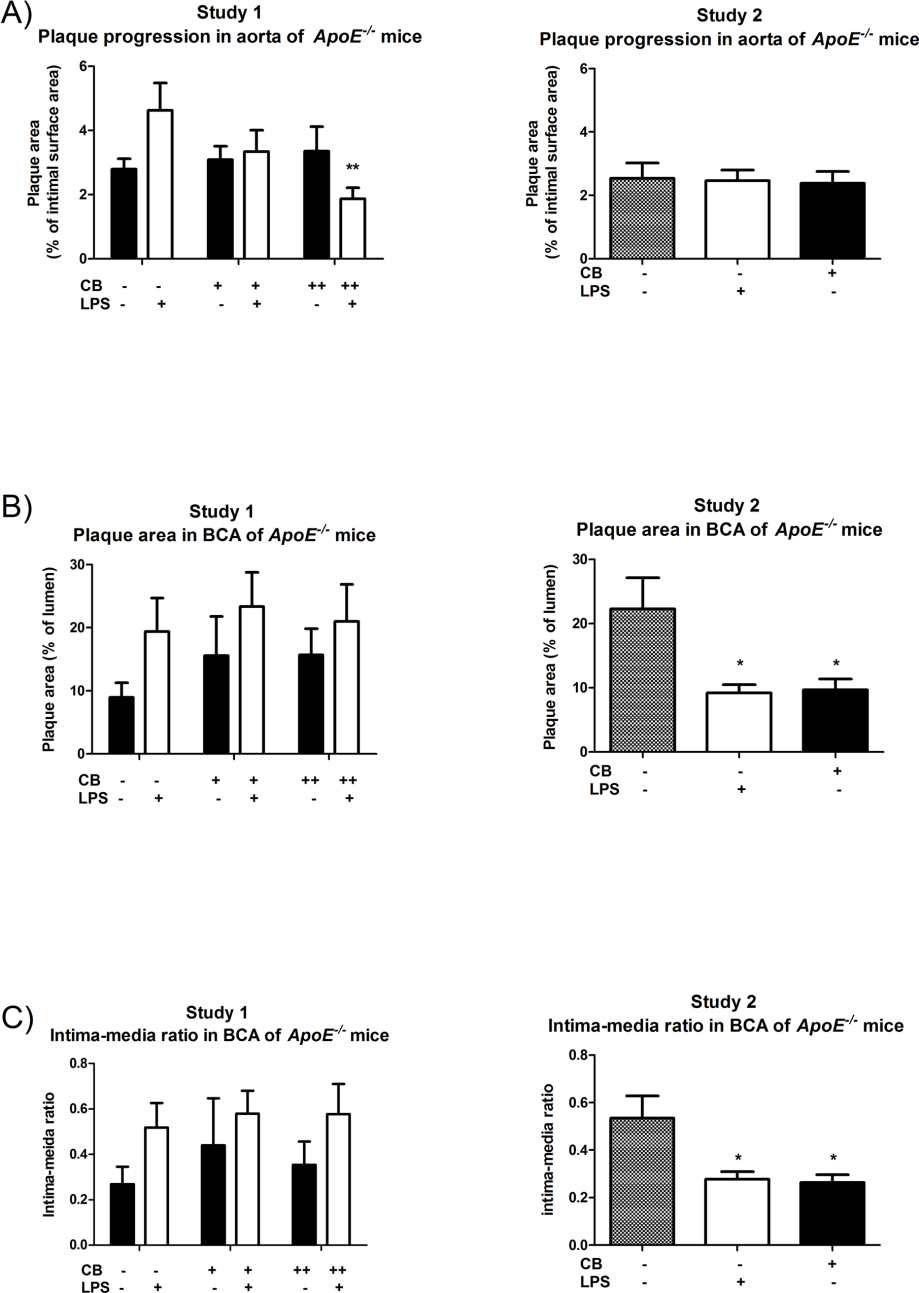
Progression of atherosclerotic plaques in the aorta and brachiocephalic artery from *ApoE*^*-/-*^ mice exposed to carbon black (CB) and/or lipopolysaccharide (LPS) by i.t. instillation. A) Atherosclerotic plaque area is expressed as the percentage of the luminal surface of the aorta covered with plaques. Calculations were made on whole aorta preparations from ascending aorta to the iliac bifurcation. B) Atherosclerotic plaque area expressed as the percentage of the lumen occupied by plaques in BCA. Six sections of BCA per animal were analyzed; three sections at 100 μm and three sections at 200 μm after the branch from the aortic arch (n = 6–10 mice per group). C) The intima-media ratio in BCA is calculated by dividing the area of the intima with the area of the media layer (n = 6–10 mice per group). Black bars represent the groups that did not receive LPS and white bars the groups that did receive LPS. Minus (-) denotes no exposure, plus denotes low (+) or high-dose (++) exposure. Data are presented as mean and SEM. Asterisks denote statistical significance *P<0.05, using one-way ANOVA with Tukey’s post-hoc test, and **P<0.01, two-factor ANOVA with Fisher’s LSD post-hoc test.

We used *en face* without staining of lipids to evaluate atherosclerotic plaque progression in aortas of *ApoE*^*-/-*^ mice (Fig 4A). In study 1 there was a statistically significant interaction between the high-dose CB+LPS group, and the LPS group (P<0.05), although there was no difference between the low-dose CB+LPS and LPS only group. However, there was 1.6-fold (95% CI: 0.9–2.7 fold) higher plaque percentage in mice only exposed to LPS compared to vehicle treated (P = 0.09, Post-hoc Fisher LSD test). For the particle only exposed mice, there were no significant differences in plaque progression between the vehicle, low-dose CB, and high-dose CB exposed mice. In study 2, there was no significant difference in plaque progression between vehicle, LPS and low-dose CB exposed mice. Pooled analysis of the results of study 1 and 2 showed no statistical significance (S2 Table).

### Unaltered progression of atherosclerosis in the BCA aorta of ApoE^-/-^ mice after repeated exposure to CB and/or LPS

There was no significant difference between the groups exposed to vehicle, low-dose CB, and high-dose CB or the groups exposed to LPS, low-dose CB+LPS, and high-dose CB+LPS (Fig 4B). In study 2 there was a statistically significant decrease in the plaque area in the BCA lumen of the LPS exposed (P<0.01), and low-dose CB exposed mice (P<0.01) as compared to the vehicle exposed mice (Fig 4B). Pooled analysis of the results of study 1 and 2 showed no statistical significance, whereas the plaque percentage was 2.8-fold (95% CI: 1.0–5.1) higher in study 2 as compared to study 1 (S2 Table).

There was no difference in intima-media ratio between the groups exposed to vehicle, low-dose CB, and high-dose CB or the groups exposed to LPS, low-dose CB + LPS, and high-dose CB+LPS in study 1 (Fig 4C). In Study 2 there was a significant decrease in the intima-media ratio in the mice exposed to LPS (P<0.05) and low-dose CB (P<0.01) compared to the vehicle exposed mice (Fig 4C). Pooled analysis of the results of study 1 and 2 showed no statistical significance, whereas there was a 3.0-fold (95% CI: 1.4–6.2 fold) higher level in study 2 as compared to study 1 (S2 Table).

The atherosclerotic lesion stage score was assessed by visual classification. S2 Fig shows plaques of present study which are considered representative of the AHA classification score [39]. Overall, there was no significant difference in the plaque morphology between the exposed groups and their respective controls (Table 2).

**Table 2:** Plaque classification score in brachiocephalic arteries from *ApoE*^*-/-*^ after repeated i.t. instillations of carbon black (CB) and/or lipopolysaccharide (LPS).

Group	Vehicle	Low-dose CB	High-dose CB	LPS	Low-dose CB+LPS	High-dose CB+LPS
Study 1	2.6± 0.6	1.8 ± 0.5	2.3 ± 0.4	2.5 ± 0.5	2.7 ± 0.5	2.2 ± 0.45
Study 2	2.2 ± 0.5	1.6 ± 0.3	ND	1.8 ± 0.3	ND	ND

The table presents the classification of atherosclerotic plaque in study 1 and 2 based on morphological characteristics according to the American Heart Association classification guideline [39]. Data in numeric numbers are presented as mean ± SEM calculated from a randomized and blinded scoring of same sections 3 times. The plaque score was not determined (ND) in all groups in study 2.

### Serum of CB-exposed ApoE^-/-^mice contains factors that cause vasoconstriction in aorta rings of unexposed wild-type mice

We investigated the response induced by plasma from *ApoE* ^*-/-*^ exposed mice (vehicle, LPS, or CB) on vasomotor activity in aorta ring segments from naïve wild-type mice using wire myography. The addition of 0.5% plasma from low-dose CB exposed mice caused contraction in aorta ring segments (6 out of 19, P<0.05, χχ^2^-test), while no contraction was observed using 0.5% plasma from the vehicle (0 out of 19) or LPS (2 out of 17) exposed mice (Table 3). When the plasma concentration was increased to 1%, contraction was observed in all three groups. The ability of endothelial-dependent vasorelaxation was preserved in all three groups after vasoconstriction in response to plasma from exposed animals.

**Table 3:** Vasoconstriction in response to vasoactive mediators in plasma from *ApoE*^*-/-*^ mice.

Group	0.5% plasma	1% plasma	Vasorelaxation (ACh)
**Vehicle**	0/19	9/19	8/9
**LPS**	2/17	4/17	4/4
**Low-dose CB**	6/19 *	10/19	8/10

The data represent the distribution of vasoconstriction and vasorelaxation in naïve aorta ring segments after adding plasma 0.5% and 1% from the vehicle, LPS, and low-dosed exposed *ApoE*^*-/-*^ to the vessel *ex vivo*. A contractility force above 0.5 mN was regarded as a significant response and a force below 0.5 mN an insignificant response. A vasorelaxation response above 50% of the maximal plasma-mediated contraction after adding 10 μM ACh was considered a significant response.

* P<0.05 using χ^2^- test.

**Fig 5:**
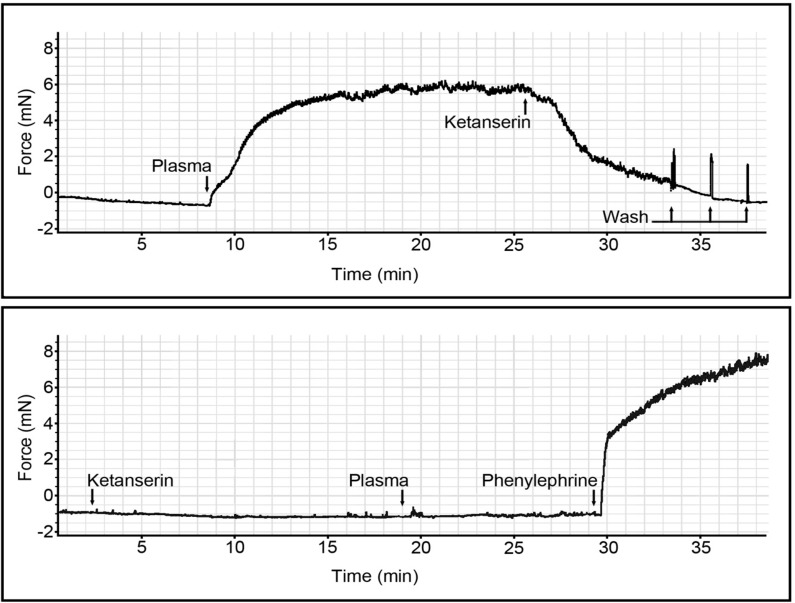
Serotonin receptor antagonist inhibition of plasma-mediated vasomotor contraction in aorta rings. The figure shows two graphical illustrations of the vasomotor function in real-time recorded in Lab Chart 8 myograph module (ADInstruments & DMT). A) 1% plasma from CB-exposed *ApoE*^*-/-*^ mice causes contraction of the naïve aorta ring segment. The addition the serotonin receptor antagonist Ketanserin (0.1 μM) on the plateau of the constriction response releases the plasma-mediated contraction. B) 17 min pre-incubation with Ketanserin (0.1 μM) inhibits the 1% plasma-mediated vessel contraction. Stimulation of the adrenergic receptors in the naïve aorta ring segments with Phenylephrine (10 μM), demonstrates that the artery has preserved the ability for receptor-mediated contraction via a receptor not specific for serotonin. The findings presented in the figure was reproduced in 3 different mice (n = 3) with the same result.

To further investigate the origin of the pharmacological modulators in the plasma, we investigated if the above-observed responses were serotonin-driven (Fig 5). The plasma mediated contraction was decreased after the addition of the serotonin receptor antagonist Ketanserin (0.1 μM). Additionally, a 15 min incubation of Ketanserin (0.1 μM) completely suppressed the plasma mediated contraction in aorta ring segments.

### The activity of LPS in the Limulus amebocyte lysate assay is inhibited by addition of CB

The observation of a statistically significant interaction between the high-dose CB and LPS on pulmonary inflammation suggested that CB may inhibit the biological activity of LPS through a physical interaction (Table 4). Firstly, the gel clot formation in the LAL assay at 4.26 μg/ml of CB indicated the presence of LPS in the sample. This was also tested with the suspensions of CB used for the *in vivo* experiments. Three 2-fold dilutions of the low dose CB+LPS suspension promoted clotting in the LAL assay (corresponding to 1.07 μg/ml of CB and 125 EU/ml of LPS). However, clotting did not occur in the high dose CB+LPS, despite the substantially higher concentration of both CB and LPS. Therefore, this suggests a CB concentration-dependent inhibition of the effects of LPS.

**Table 4:** Interaction between carbon black (CB) and lipopolysaccharide (LPS).

**CB dilution (μg/ml)**										
Replicate	25.6	12.8	8.3	6.4	4.3	3.2	2.1	1.1	0.5	LPS-free water
1	+	+	+	+	+	-	-	-	-	-
2	+	+	+	+	+	+	-	-	-	-
**Low-dose CB+LPS dilution (μg/ml) / (EU/ml)**										
Replicate	8.5 / 1000	4.3 / 500	2.1 / 250	1.1 / 125	0.5 / 62.5	LPS-free water				
1	+	+	+	+	-	-				
2	+	+	+	+	-	-				
**High-dose CB+LPS dilution (μg/mL) / (EU/ml)**										
Replicate	25.6 / 1000	12.8 / 500	6.4 / 250	3.2 / 125	1.6 / 62.5	LPS-free water				
1	-	-	-	-	-	-				
2	-	-	-	-	-	-				

Nanosized CB was sonicated in LPS free water and diluted in LPS free water to the concentration used for *in vivo* study exposure (the highest concentration of low-dose (8.5 μg/mouse), and high-dose (25.6 μg/mouse)). Each assay was performed in duplicates. “+” indicates a clot equals LPS in the sample, and “–”indicates no-clot that is equal to no LPS in the sample.

## Discussion

In the present study, we investigated the *in vivo* pulmonary and cardiovascular effects following repeated exposure to nanosized CB and/or LPS. Our findings show that repeated pulmonary exposure to CB increases the total cell number in BALF with a modest yet significant influx of neutrophils. The data are in concordance with results from previous studies on repeated i.t. instillation of Printex 90 to Balb/c mice [45]. It has also been shown that multiple i.t. instillations of LPS increased the influx of neutrophils in BALF [46]. We observed a similar pattern following 10 weeks of pulmonary exposure to LPS, regardless of co-exposure to CB. The total BALF cell number in the LPS exposed groups was significantly increased and mainly driven by the strong neutrophil influx contributing to approximately 50% of the total cell number. Moreover, the macrophage numbers were significantly increased in the LPS- and low-dose CB+LPS group, but not the high-dose CB+LPS group following repeated exposures. We only found increased expression of *Saa3* in the lungs of mice exposed to high-dose CB+LPS; this is different from observations in studies with a single i.t. instillation of nanosized CB, which increased *Saa3* expression levels in the lungs of C57BL/6 mice at day 1, 3 and 28 post-exposure [36]. The differences in *Saa3* expression levels witnessed here and previous studies might be due to variances in the acute phase response between single and repeated exposures (i.e. high levels of *Saa3* after a single exposure). If inflammation persists, it may lead to a redox imbalance favoring a pro-oxidant milieu causing the depletion of antioxidant enzymes [47]. It is recognized that altered glutathione metabolism occurs in inflammatory lung diseases [48]. Nevertheless, the levels and balance of total and reduced glutathione in lung tissue of *ApoE*^*-/-*^ mice at 24 h after last exposure were not affected, indicating that the pulmonary exposure did not cause oxidative stress in the lungs (or it was too late to investigate this end-point). It is our experience that it requires relatively high bolus doses of NMs to cause glutathione depletion in the lungs; e.g. i.t. instillation of 15 μg ZnO caused both cytotoxicity and depletion of glutathione in mouse lung [49]. However, it should also be noted that the lung tissue samples had a relatively high content of oxidized glutathione (approximately 30%), which may indicate spontaneous oxidation of the samples during storage at -80°C. The repeated exposure of CB and/or LPS may have been associated with subtle differences in the glutathione status, but not an adaptation in terms of increased glutathione levels in the lungs.

The exposure to CB did not accelerate plaque progression in the aorta or the BCA. An earlier study on repeated exposure to CB showed that a much higher dose administered by i.t. instillation (1000 μg/mouse per week for ten weeks) was associated with increased plaque progression in LDL receptor knockout mice on a cholesterol-rich diet [9]. It is well documented that cholesterol-enriched diet *per se* accelerates plaque progression in both *ApoE*^*-/-*^ and LDL receptor knockout mice [50]. However, it is not possible to disentangle the effect of high CB dose from diet-induced susceptibility to plaque progression. In an earlier study, we exposed 48–49 weeks old *ApoE*^*-/-*^ mice to Printex 90 by i.t. instillation (0.5 mg/kg once a week for 2 weeks), but the study was terminated prematurely because of a high mortality rate after each round of exposure [10].

Pulmonary exposure to LPS in study 1 indicated a tendency towards accelerated plaque progression in the aorta; therefore, study 2 was carried out to increase the statistical power. However, the results of study 2 did not support the initial findings of study 1. Previous studies have indicated that LPS exposure by intraperitoneal injection was associated with increased plaque progression in the aorta [51, 52]. Surprisingly, we found that mice exposed to high-dose CB+LPS had lower plaque progression and less pronounced pulmonary inflammation as compared to the LPS exposed group. This led us to investigate if CB could inhibit the effect of LPS using the LAL assay. As shown in Table 4 the high dose CB was able to inhibit gel clot formation i.e. the LPS-mediated effect, whereas low-dose CB+LPS did not. Indeed, the LAL assay showed that 1 μg CB was enough to eliminate the effect of at least 39 endotoxin units. However, clotting indicated that LPS was present in CB in concentrations down to 4.3 μg/ml. Thus, it suggests that CB affects the action of LPS and its downstream signaling cascade. One possible explanation for this inhibition could be the adsorption of LPS by CB [53].

Endothelial dysfunction, including impaired vasomotor function, is a hallmark of early progression of atherosclerosis [54]. Vasomotor dysfunction also occurs by pulmonary exposure to particles in wild-type and atherosclerosis-prone animals as well as in humans with clinical manifestation of atherosclerosis [4]. Although this may be related to structural or molecular changes in the endothelium or intima, recent evidence also indicates that pulmonary toxicity can be conveyed systemically with circulating vasoactive molecules capable of modulating vasomotor function in arteries from naïve animals [18–20]. Thus, we hypothesized that vasomotor active molecules in the plasma from CB or LPS exposed *ApoE*^*-/-*^ mice would alter the vasomotor function in aortas from naïve C57BL/6 mice. There was an increased vasoconstriction response in aorta rings that were incubated with 0.5% plasma from the low-dose CB exposed mice. Increasing the concentration to 1% caused a contraction in all three groups. This effect was attributed to increased level of serotonin in plasma of the exposed mice because incubation with ketanserin abolished the vasoconstriction response. The antihypertensive action of ketanserin in humans has been ascribed to its action on both the S_2_-serotoninergic and α_1_-adrenergic receptors [44]. However, it has been shown that the *ex vivo* contractile response of arteries to serum was abolished in the presence of ketanserin, whereas the α_1_-adrenergic receptor prazosin had no effect [55]. Extended *ex vivo* cultures of arteries in the presence of serum for 4 days also produced a progressive constriction and arterial wall remodeling, which was not released by treatment with ketanserin, indicating a different mechanism of constriction than soluble vasoconstrictors in long-term cultures [56]. Unfortunately, we did not have sufficient quantities of plasma to measure the serotonin concentration. The concentration of serotonin in plasma is lower than serum; e.g. studies that have assessed serotonin concentration in samples from the same individuals have shown 3–15 versus 72–137 ng/ml in plasma and serum, respectively [57, 58]. The higher serotonin concentration in serum could be related to release during the blood clot formation, which may cause degranulation of platelets. Indeed, it does seem that incubation with serum had a strong constriction response in other studies; e.g. 1% serum produced a constriction response in aorta rings that was comparable to that induced by KPSS [14]. Other observations from the same group showed that aorta rings from unexposed mice had a basal tonus of 8.8 mN and addition of 2.5% serum increased it with 5.3 mN [20]. In comparison, the basal tonus was approximately 5 mN in aorta segments and addition of 0.5% plasma from CB-exposed mice produced approximately 40% increased tonus (i.e. a net increase of 2 mN). It is possible that the elevated serotonin concentrations in the plasma originate from platelets as either a cause or consequence of prothrombotic propensity (direct systemic administration of CB in mice has demonstrated to increase prothrombotic activity) [59]. In addition, i.t. instillation of CB in rats has resulted in platelet hyperactivity [60], although other studies showed no effect on plasma levels of coagulation factors or infarct size after cardiac ischemic/reperfusion injury [61–63]. An increased plasma concentration of serotonin might be a contributing factor to hypertension in humans due to increased vasoconstriction. Exposure to particulate matter in air pollution is associated with hypertension [64]. Interestingly, a panel study with personal black carbon measurements (i.e. a proxy-measure of air pollution) showed association between exposure and rapid changes in carotid arterial stiffening [65]. Increased arterial stiffening is a predictor of cardiovascular disease mortality in patients with essential hypertension [66]. Moreover, it has also been shown that inhalation exposure to concentrated ambient air particulate matter in *ApoE*^*-/-*^ mice augmented the vasoconstrictor response to serotonin in aorta rings [67].

## Conclusion

This study shows that 10 weeks of i.t. instillation with nanosized CB, LPS or a combination in *ApoE*^*-/-*^ mice induced pulmonary inflammation that was mainly characterized by an influx of neutrophils. The lung antioxidant defense (glutathione) and pulmonary expression of *Saa3* were unaffected by repeated exposures. We did not find evidence of accelerated progression of atherosclerosis in the aorta or BCA after exposure to CB or LPS. Nevertheless, plasma from *ApoE*^*-/-*^ mice exposed to CB caused vasoconstriction when added to aorta rings from naïve wild-type mice, which appeared to be related to increased plasma levels of serotonin.

## Supporting Information

S1 Fig Dynamic Light Scattering measurements number distribution of nanosized CB suspended in nanopure water. A) Low dose CB (170 μg/ml), average PDI = 0.18, average size = 44 nm. B) Low dose CB (170 μg/ml) spiked with LPS (2 μg/ml), average PDI = 0.35, average size = 1281 nm. C) High dose CB (512 μg/ml), average PDI = 0.25, average size = 38 nm. High dose CB (512 μg/ml) spiked with LPS (2 μg/ml), average PDI = 0.57, average size = 1718 nm.
(DOCX)

S2 Fig **Standardized mean difference (SMD) in total cells (top) or neutrophils (bottom) in BALF as a function of the dose of Printex 90 administered by intratracheal instillation in C57BL/6 mice (0.67, 2.6, 18, 54 or 162 μg/mouse).** The animals were sacrificed at 24 h post-exposure. The SMD has been calculated using Review Manager (RevMan) version 5.0 (The Nordic Cochrane Centre. The Cochrane Collaboration. 2008). The SMD is the difference between the two groups divided by the pooled standard deviation. The SMD in the top and bottom graphs cannot be compared with nominal values (i.e. number of cells) because they represent different scales. The SMD for total cells is close to zero (i.e. no effect), whereas there is a slightly increased influx of neutrophils at the low doses. The dose of 18 μg/mouse shows increased total cells as compared to low doses and a similar level of neutrophils. The responses at doses 54 and 162 μg/mouse suggest a plateau for both total cells and neutrophils in BALF.
(DOCX)

S3 Fig Representative sections of BCA from the *ApoE*^*-/-*^ mice. The sections were stained with Masson’s trichrome stain. Classification of atherosclerotic lesion was based on guidelines from American Heart Association. Stages I-III are clinically silent lesions and precursors to advanced lesions. Stage IV is an advanced lesions called atheroma and have a core of accumulated extracellular lipid. Stage V represents advanced lesions called fibroatheroma lesions and has multiple lipid cores, fibrotic layers and calcifications (Stary et al. 1995).
(DOCX)

S1 Table BALF cell number and distribution 24 h post-exposure to LPS. Upper table shows the BALF cell number and distribution 24 h after a single exposure to LPS. Lower table shows the BALF cell distribution post 24.h after last exposure (one i.t. instillations once a week for 4 weeks). Asterisk denote ***P<0.001. **P<0.01 and *P<0.05 cells influx in exposed group compared to vehicle group. Data are presented as mean ± SEM. Statistical analyses were performed using one-way ANOVA with Tukey’s post-hoc test.
(DOCX)

S2 Table Atherosclerosis in the BCA of *ApoE*^*-/-*^ mice at 24 h post-exposure. (DOCX)

## Abbreviations

Ach: Acetylcholine
AHA: American Heart Association
ApoE: Apolipoprotein E
BAL: Bronchoalveolar lavage
BCA: Brachiocephalic artery
CB: carbon black
EDTA: Ethylenediaminetetraacetic acid
GSSG: Glutathione disulphide
I.t.: Intratracheal instillation
K-PSS: Potassium physiological saline solution
LAL: Limulus amebocyte lysate
LDL: Low density lipoprotein
LPS: Lipopolysaccharide
LSD: Least statistical differences
NM: Nanomaterial
PSS: Physiological saline solution
qPCR: quantitative-PCR
Saa3: Serum Amyloid A 3

## References

[1] Lim SS, Vos T, Flaxman AD, Danaei G, Shibuya K, Adair-Rohani H, et al A comparative risk assessment of burden of disease and injury attributable to 67 risk factors and risk factor clusters in 21 regions, 1990–2010: a systematic analysis for the Global Burden of Disease Study 2010. Lancet 2012, 380:2224–2260. 10.1016/S0140-6736(12)61766-8 23245609 PMC4156511

[2] Araujo JA, Nel AE. Particulate matter and atherosclerosis: role of particle size, composition and oxidative stress. Part Fibre Toxicol 2009, 6:24 10.1186/1743-8977-6-24 19761620 PMC2761850

[3] Brook RD, Rajagopalan S, Pope CA III, Brook JR, Bhatnagar A, Diez-Roux AV, et al Particulate matter air pollution and cardiovascular disease: An update to the scientific statement from the American Heart Association. Circulation 2010, 121:2331–2378. 10.1161/CIR.0b013e3181dbece1 20458016

[4] Møller P, Mikkelsen L, Vesterdal LK, Folkmann JK, Forchhammer L, Roursgaard M, et al Hazard identification of particulate matter on vasomotor dysfunction and progression of atherosclerosis. Crit Rev Toxicol 2011, 41:339–368. 10.3109/10408444.2010.533152 21345153

[5] Saber AT, Jacobsen NR, Jackson P, Poulsen SS, Kyjovska ZO, Halappanavar S, et al Particle-induced pulmonary acute phase response may be the causal link between particle inhalation and cardiovascular disease. Wiley Interdiscip Rev Nanomed Nanobiotechnol 2014, 6:517–531. 10.1002/wnan.1279 24920450 PMC4285160

[6] Kido T, Tamagawa E, Bai N, Suda K, Yang HH, Li Y, et al Particulate matter induces translocation of IL-6 from the lung to the systemic circulation. Am J Respir Cell Mol Biol 2011, 44:197–204. 10.1165/rcmb.2009-0427OC 20378751

[7] Nurkiewicz TR, Porter DW, Barger M, Millecchia L, Rao KM, Marvar PJ, et al Systemic microvascular dysfunction and inflammation after pulmonary particulate matter exposure. Environ Health Perspect 2006, 114:412–419. 16507465 10.1289/ehp.8413 PMC1392236

[8] Brown DM, Wilson MR, MacNee W, Stone V, Donaldson K. Size-dependent proinflammatory effects of ultrafine polystyrene particles: a role for surface area and oxidative stress in the enhanced activity of ultrafines. Toxicol Appl Pharmacol 2001, 175:191–199. 11559017 10.1006/taap.2001.9240

[9] Niwa Y, Hiura Y, Murayama T, Yokode M, Iwai N. Nano-sized carbon black exposure exacerbates atherosclerosis in LDL-receptor knockout mice. Circ J 2007, 71(7):1157–1161. 17587728 10.1253/circj.71.1157

[10] Vesterdal LK, Folkmann JK, Jacobsen NR, Sheykhzade M, Wallin H, Loft S, et al Pulmonary exposure to carbon black nanoparticles and vascular effects. Part Fibre Toxicol 2010, 7:33 10.1186/1743-8977-7-33 21054825 PMC2991279

[11] Kim JK, Kang MG, Cho HW, Han JH, Chung YH, Rim KT, et al Effect of Nano-sized Carbon Black Particles on Lung and Circulatory System by Inhalation Exposure in Rats. Saf Health Work 2011, 2:282–289. 10.5491/SHAW.2011.2.3.282 22953212 PMC3430899

[12] Courtois A, Andujar P, Ladeiro Y, Baudrimont I, Delannoy E, Leblais V, et al Impairment of NO-dependent relaxation in intralobar pulmonary arteries: comparison of urban particulate matter and manufactured nanoparticles. Environ Health Perspect 2008, 116:1294–1299. 10.1289/ehp.11021 18941568 PMC2569085

[13] Folkmann JK, Vesterdal LK, Sheykhzade M, Loft S, Møller P. Endothelial dysfunction in normal and prediabetic rats with metabolic syndrome exposed by oral gavage to carbon black nanoparticles. Toxicol Sci 2012, 129:98–107. 10.1093/toxsci/kfs180 22610611

[14] Aragon MJ, Chrobak I, Brower J, Roldan L, Fredenburgh LE, McDonald JD, et al Inflammatory and Vasoactive Effects of Serum Following Inhalation of Varied Complex Mixtures. Cardiovasc Toxicol 2016, 16:163–171. 10.1007/s12012-015-9325-z 25900702 PMC4618267

[15] Vesterdal LK, Mikkelsen L, Folkmann JK, Sheykhzade M, Cao Y, Roursgaard M, et al Carbon black nanoparticles and vascular dysfunction in cultured endothelial cells and artery segments. Toxicol Lett 2012, 214:19–26. 10.1016/j.toxlet.2012.07.022 22885096

[16] Kermanizadeh A, Balharry D, Wallin H, Loft S, Møller P. Nanomaterial translocation—the biokinetics, tissue accumulation, toxicity and fate of materials in secondary organs—a review. Crit Rev Toxicol 2015, 45:837–872. 10.3109/10408444.2015.1058747 26140391

[17] Møller P, Christophersen DV, Jacobsen NR, Skovmand A, Gouveia AC, Andersen MH, et al Atherosclerosis and vasomotor dysfunction in arteries of animals after exposure to combustion-derived particulate matter or nanomaterials. Crit Rev Toxicol 2016, 46:437–476. 10.3109/10408444.2016.1149451 27028752

[18] Aragon MJ, Chrobak I, Brower J, Roldan L, Fredenburgh LE, McDonald JD, et al Inflammatory and Vasoactive Effects of Serum Following Inhalation of Varied Complex Mixtures. Cardiovasc Toxicol 2016, 163–171. 10.1007/s12012-015-9325-z 25900702 PMC4618267

[19] Paffett ML, Zychowski KE, Sheppard L, Robertson S, Weaver JM, Lucas SN, et al Ozone Inhalation Impairs Coronary Artery Dilation via Intracellular Oxidative Stress: Evidence for Serum-Borne Factors as Drivers of Systemic Toxicity. Toxicol Sci 2015, 146:244–253. 10.1093/toxsci/kfv093 25962394 PMC4607748

[20] Robertson S, Colombo ES, Lucas SN, Hall PR, Febbraio M, Paffett ML, et al CD36 mediates endothelial dysfunction downstream of circulating factors induced by O3 exposure. Toxicol Sci 2013, 134:304–311. 10.1093/toxsci/kft107 23650127 PMC3707435

[21] Jacobsen NR, Møller P, Jensen KA, Vogel U, Ladefoged O, Loft S, et al Lung inflammation and genotoxicity following pulmonary exposure to nanoparticles in ApoE-/- mice. Part Fibre Toxicol 2009, 6:2 10.1186/1743-8977-6-2 19138394 PMC2636756

[22] Gitlin JM, Loftin CD. Cyclooxygenase-2 inhibition increases lipopolysaccharide-induced atherosclerosis in mice. Cardiovasc Res 2009, 81:400–407. 10.1093/cvr/cvn286 18948273 PMC2639107

[23] Bourdon JA, Halappanavar S, Saber AT, Jacobsen NR, Williams A, Wallin H, et al Hepatic and pulmonary toxicogenomic profiles in mice intratracheally instilled with carbon black nanoparticles reveal pulmonary inflammation, acute phase response, and alterations in lipid homeostasis. Toxicol Sci 2012, 127:474–484. 10.1093/toxsci/kfs119 22461453 PMC3355316

[24] Bourdon JA, Saber AT, Halappanavar S, Jackson PA, Wu D, Hougaard KS, et al Carbon black nanoparticle intratracheal installation results in large and sustained changes in the expression of miR-135b in mouse lung. Environ Mol Mutagen 2012, 53:462–468. 10.1002/em.21706 22753103

[25] Bourdon JA, Saber AT, Jacobsen NR, Jensen KA, Madsen AM, Lamson JS, et al Carbon black nanoparticle instillation induces sustained inflammation and genotoxicity in mouse lung and liver. Part Fibre Toxicol 2012, 9:5 10.1186/1743-8977-9-5 22300514 PMC3293019

[26] Hogsberg T, Jacobsen NR, Clausen PA, Serup J. Black tattoo inks induce reactive oxygen species production correlating with aggregation of pigment nanoparticles and product brand but not with the polycyclic aromatic hydrocarbon content. Exp Dermatol 2013, 22:464–469. 10.1111/exd.12178 23800057

[27] Jacobsen NR, Pojana G, White P, Møller P, Cohn CA, Korsholm KS, et al Genotoxicity, cytotoxicity, and reactive oxygen species induced by single-walled carbon nanotubes and C(60) fullerenes in the FE1-Mutatrade markMouse lung epithelial cells. Environ Mol Mutagen 2008, 49:476–487. 10.1002/em.20406 18618583

[28] Jacobsen NR, White PA, Gingerich J, Møller P, Saber AT, Douglas GR, et al Mutation spectrum in FE1-MUTA(TM) Mouse lung epithelial cells exposed to nanoparticulate carbon black. Environ Mol Mutagen 2011, 52:331–337. 10.1002/em.20629 20963790

[29] Kyjovska ZO, Jacobsen NR, Saber AT, Bengtson S, Jackson P, Wallin H, et al U: DNA strand breaks, acute phase response and inflammation following pulmonary exposure by instillation to the diesel exhaust particle NIST1650b in mice. Mutagenesis 2015, 30:499–507. 10.1093/mutage/gev009 25771385

[30] Møller P, Jacobsen NR, Folkmann JK, Danielsen PH, Mikkelsen L, Hemmingsen JG, et al Role of oxidative damage in toxicity of particulates. Free Radic Res 2010, 44:1–46. 10.3109/10715760903300691 19886744

[31] Jacobsen NR, Saber AT, White P, Møller P, Pojana G, Vogel U, et al Increased mutant frequency by carbon black, but not quartz, in the lacZ and cII transgenes of muta mouse lung epithelial cells. Environ Mol Mutagen 2007, 48:451–461. 17584883 10.1002/em.20300

[32] Saber AT, Jensen KA, Jacobsen NR, Birkedal R, Mikkelsen L, Møller P, et al Inflammatory and genotoxic effects of nanoparticles designed for inclusion in paints and lacquers. Nanotoxicology 2012, 6:453–471. 10.3109/17435390.2011.587900 21649461

[33] Saber AT, Koponen IK, Jensen KA, Jacobsen NR, Mikkelsen L, Møller P, et al Inflammatory and genotoxic effects of sanding dust generated from nanoparticle-containing paints and lacquers. Nanotoxicology 2012, 6:776–788. 10.3109/17435390.2011.620745 21995293

[34] Jackson P, Hougaard KS, Boisen AM, Jacobsen NR, Jensen KA, Møller P, et al Pulmonary exposure to carbon black by inhalation or instillation in pregnant mice: effects on liver DNA strand breaks in dams and offspring. Nanotoxicology 2012, 6:486–500. 10.3109/17435390.2011.587902 21649560 PMC3411122

[35] Senft AP, Dalton TP, Shertzer HG. Determining glutathione and glutathione disulfide using the fluorescence probe o-phthalaldehyde. Anal Biochem 2000, 280:80–86. 10805524 10.1006/abio.2000.4498

[36] Saber AT, Lamson JS, Jacobsen NR, Ravn-Haren G, Hougaard KS, et al Particle-induced pulmonary acute phase response correlates with neutrophil influx linking inhaled particles and cardiovascular risk. PLoS One 2013, 8:e69020 10.1371/journal.pone.0069020 23894396 PMC3722244

[37] Saber AT, Halappanavar S, Folkmann JK, Bornholdt J, Boisen AM, Møller P, et al Lack of acute phase response in the livers of mice exposed to diesel exhaust particles or carbon black by inhalation. Part Fibre Toxicol 2009, 6:12 10.1186/1743-8977-6-12 19374780 PMC2673201

[38] Livak KJ, Schmittgen TD. Analysis of relative gene expression data using real-time quantitative PCR and the 2(-Delta Delta C(T)) Method. Methods 2001, 25:402–408. 11846609 10.1006/meth.2001.1262

[39] Stary HC, Chandler AB, Dinsmore RE, Fuster V, Glagov S, Insull W Jr, et al A definition of advanced types of atherosclerotic lesions and a histological classification of atherosclerosis. A report from the Committee on Vascular Lesions of the Council on Arteriosclerosis, American Heart Association. Circulation 1995, 92:1355–1374. 7648691 10.1161/01.cir.92.5.1355

[40] Hansen CS, Sheykhzade M, Møller P, Folkmann JK, Amtorp O, Jonassen T, et al Diesel exhaust particles induce endothelial dysfunction in apoE-/- mice. Toxicol Appl Pharmacol 2007, 219:24–32. 17234226 10.1016/j.taap.2006.10.032

[41] Mikkelsen L, Sheykhzade M, Jensen KA, Saber AT, Jacobsen NR, Vogel U, et al Modest effect on plaque progression and vasodilatory function in atherosclerosis-prone mice exposed to nanosized TiO(2). Part Fibre Toxicol 2011, 8:32 10.1186/1743-8977-8-32 22074227 PMC3245428

[42] Vesterdal LK, Folkmann JK, Jacobsen NR, Sheykhzade M, Wallin H, Loft S, et al Modest vasomotor dysfunction induced by low doses of C60 fullerenes in apolipoprotein E knockout mice with different degree of atherosclerosis. Part Fibre Toxicol 2009, 6:5 10.1186/1743-8977-6-5 19243580 PMC2672923

[43] Vesterdal LK, Jantzen K, Sheykhzade M, Roursgaard M, Folkmann JK, Loft S, et al Pulmonary exposure to particles from diesel exhaust, urban dust or single-walled carbon nanotubes and oxidatively damaged DNA and vascular function in apoE(-/-) mice. Nanotoxicology 2014, 8:61–71. 10.3109/17435390.2012.750385 23148895

[44] Vanhoutte P, Amery A, Birkenhager W, Breckenridge A, Buhler F, Distler A, et al Serotoninergic mechanisms in hypertension. Focus on the effects of ketanserin. Hypertension 1988, 11:111–133. 3277910 10.1161/01.hyp.11.2.111

[45] Shwe TT, Yamamoto S, Kakeyama M, Kobayashi T, Fujimaki H. Effect of intratracheal instillation of ultrafine carbon black on proinflammatory cytokine and chemokine release and mRNA expression in lung and lymph nodes of mice. Toxicol Appl Pharmacol 2005, 209:51–61. 16331831 10.1016/j.taap.2005.03.014

[46] Corbel M, Theret N, Caulet-Maugendre S, Germain N, Lagente V, Clement B, et al Repeated endotoxin exposure induces interstitial fibrosis associated with enhanced gelatinase (MMP-2 and MMP-9) activity. Inflamm Res 2001, 50:129–135. 11339500 10.1007/s000110050736

[47] Møller P, Christophersen DV, Jensen DM, Kermanizadeh A, Roursgaard M, Jacobsen NR, et al Role of oxidative stress in carbon nanotube-generated health effects. Arch Toxicol 2014, 88:1939–1964. 10.1007/s00204-014-1356-x 25212906

[48] Lee KS, Kim SR, Park HS, Park SJ, Min KH, Lee KY, et al A novel thiol compound, N-acetylcysteine amide, attenuates allergic airway disease by regulating activation of NF-kappaB and hypoxia-inducible factor-1alpha. Exp Mol Med 2007, 39:756–768. 18160846 10.1038/emm.2007.82

[49] Jacobsen NR, Stoeger T, van den Brule S, Saber AT, Beyerle A, Vietti G, et al Acute and subacute pulmonary toxicity and mortality in mice after intratracheal instillation of ZnO nanoparticles in three laboratories. Food Chem Toxicol 2015, 85:84–95. 10.1016/j.fct.2015.08.008 26260750

[50] Kennedy AJ, Ellacott KL, King VL, Hasty AH. Mouse models of the metabolic syndrome. Dis Model Mech 2010, 3:156–166. 10.1242/dmm.003467 20212084 PMC2869491

[51] Andoh Y, Ogura H, Satoh M, Shimano K, Okuno H, Fujii S, et al Natural killer T cells are required for lipopolysaccharide-mediated enhancement of atherosclerosis in apolipoprotein E-deficient mice. Immunobiology 2013, 218:561–569. 10.1016/j.imbio.2012.07.022 22954709 PMC3535521

[52] Ostos MA, Recalde D, Zakin MM, Scott-Algara D. Implication of natural killer T cells in atherosclerosis development during a LPS-induced chronic inflammation. FEBS letters 2002, 519:23–29. 12023012 10.1016/s0014-5793(02)02692-3

[53] Cai X, Ramalingam R, Wong HS, Cheng J, Ajuh P, Cheng SH, et al Characterization of carbon nanotube protein corona by using quantitative proteomics. Nanomedicine 2013, 9:583–593. 10.1016/j.nano.2012.09.004 23117048

[54] Libby P, Ridker PM, Hansson GK. Progress and challenges in translating the biology of atherosclerosis. Nature 2011, 473:317–325. 10.1038/nature10146 21593864

[55] De Mey JG, Uitendaal MP, Boonen HC, Vrijdag MJ, Daemen MJ, Struyker-Boudier HA. Acute and long-term effects of tissue culture on contractile reactivity in renal arteries of the rat. Circ Res 1989, 65:1125–1135. 2791222 10.1161/01.res.65.4.1125

[56] Bakker EN, van Der Meulen ET, Spaan JA, VanBavel E. Organoid culture of cannulated rat resistance arteries: effect of serum factors on vasoactivity and remodeling. American Journal of Physiology Heart and Circulatory Physiology 2000, 278:H1233–1240. 10749719 10.1152/ajpheart.2000.278.4.H1233

[57] Koch DD, Kissinger PT. Determination of serotonin in serum and plasma by liquid chromatography with precolumn sample enrichment and electrochemical detection. Analytical Chemistry 1980, 52:27–29. 7356172 10.1021/ac50051a008

[58] Lee GS, Simpson C, Sun BH, Yao C, Foer D, Sullivan B, et al Measurement of plasma, serum, and platelet serotonin in individuals with high bone mass and mutations in LRP5. Journal of Bone and Mineral Research: the official journal of the American Society for Bone and Mineral Research 2014, 29:976–981. 10.1002/jbmr.2086 PMC3935983 24038240

[59] Holzer M, Bihari P, Praetner M, Uhl B, Reichel C, Fent J, et al Carbon-based nanomaterials accelerate arteriolar thrombus formation in the murine microcirculation independently of their shape. Journal of Applied Toxicology 2014, 34:1167–1176. 10.1002/jat.2996 24531921

[60] Kim H, Oh SJ, Kwak HC, Kim JK, Lim CH, Yang JS, et al The impact of intratracheally instilled carbon black on the cardiovascular system of rats: elevation of blood homocysteine and hyperactivity of platelets. J Toxicol Environ Health A 2012, 75:1471–1483. 10.1080/15287394.2012.722519 23116452

[61] Gilmour PS, Ziesenis A, Morrison ER, Vickers MA, Drost EM, Ford I,. et al Pulmonary and systemic effects of short-term inhalation exposure to ultrafine carbon black particles. Toxicol Appl Pharmacol 2004, 195:35–44. 14962503 10.1016/j.taap.2003.10.003

[62] Harder V, Gilmour P, Lentner B, Karg E, Takenaka S, Ziesenis A, et al Cardiovascular responses in unrestrained WKY rats to inhaled ultrafine carbon particles. Inhal Toxicol 2005, 17:29–42. 15764481 10.1080/08958370590885681

[63] Tong H, McGee JK, Saxena RK, Kodavanti UP, Devlin RB, Gilmour MI. Influence of acid functionalization on the cardiopulmonary toxicity of carbon nanotubes and carbon black particles in mice. Toxicol Appl Pharmacol 2009, 239:224–232. 10.1016/j.taap.2009.05.019 19481103

[64] Fuks KB, Weinmayr G, Foraster M, Dratva J, Hampel R, Houthuijs D, et al Arterial blood pressure and long-term exposure to traffic-related air pollution: an analysis in the European Study of Cohorts for Air Pollution Effects (ESCAPE). Environ Health Perspect 2014, 122:896–905. 10.1289/ehp.1307725 24835507 PMC4154218

[65] Provost EB, Louwies T, Cox B, Op't Roodt J, Solmi F, Dons E, et al Short-term fluctuations in personal black carbon exposure are associated with rapid changes in carotid arterial stiffening. Environment International 2016, 88:228–234. 10.1016/j.envint.2015.12.023 26773393

[66] Laurent S, Boutouyrie P, Asmar R, Gautier I, Laloux B, Guize L, et al Aortic stiffness is an independent predictor of all-cause and cardiovascular mortality in hypertensive patients. Hypertension 2001, 37:1236–1241. 11358934 10.1161/01.hyp.37.5.1236

[67] Sun Q, Wang A, Jin X, Natanzon A, Duquaine D, Brook RD, et al Long-term air pollution exposure and acceleration of atherosclerosis and vascular inflammation in an animal model. JAMA 2005, 294:3003–3010. 16414948 10.1001/jama.294.23.3003

