# Supplementary material for: Inflammation and Vascular Effects after Repeated Intratracheal Instillations of Carbon Black and Lipopolysaccharide
Source: PLoS One. 2016 Aug 29;11(8):e0160731. doi: 10.1371/journal.pone.0160731 (PMC5003393; doi:10.1371/journal.pone.0160731)
Supplement: S1 Fig — A) Low dose CB (170 μg/ml), average PDI = 0.18, average size = 44 nm. B) Low dose CB (170 μg/ml) spiked with LPS (2 μg/ml), average PDI = 0.35, average size = 1281 nm. C) High dose CB (512 μg/ml), average PDI = 0.25, average size = 38 nm. High dose CB (512 μg/ml) spiked with LPS (2 μg/ml), average PDI = 0.57, average size = 1718 nm. (DOCX) [file pone.0160731.s001.docx]

**A) Low dose CB**

**B) Low dose CB spiked with LPS**

**C) High dose CB**

**D) High dose CB spiked with LPS**
