# Supplementary figures and images for: Inflammation and Vascular Effects after Repeated Intratracheal Instillations of Carbon Black and Lipopolysaccharide
Source: PLoS One. 2016 Aug 29;11(8):e0160731. doi: 10.1371/journal.pone.0160731 (PMC5003393; doi:10.1371/journal.pone.0160731)

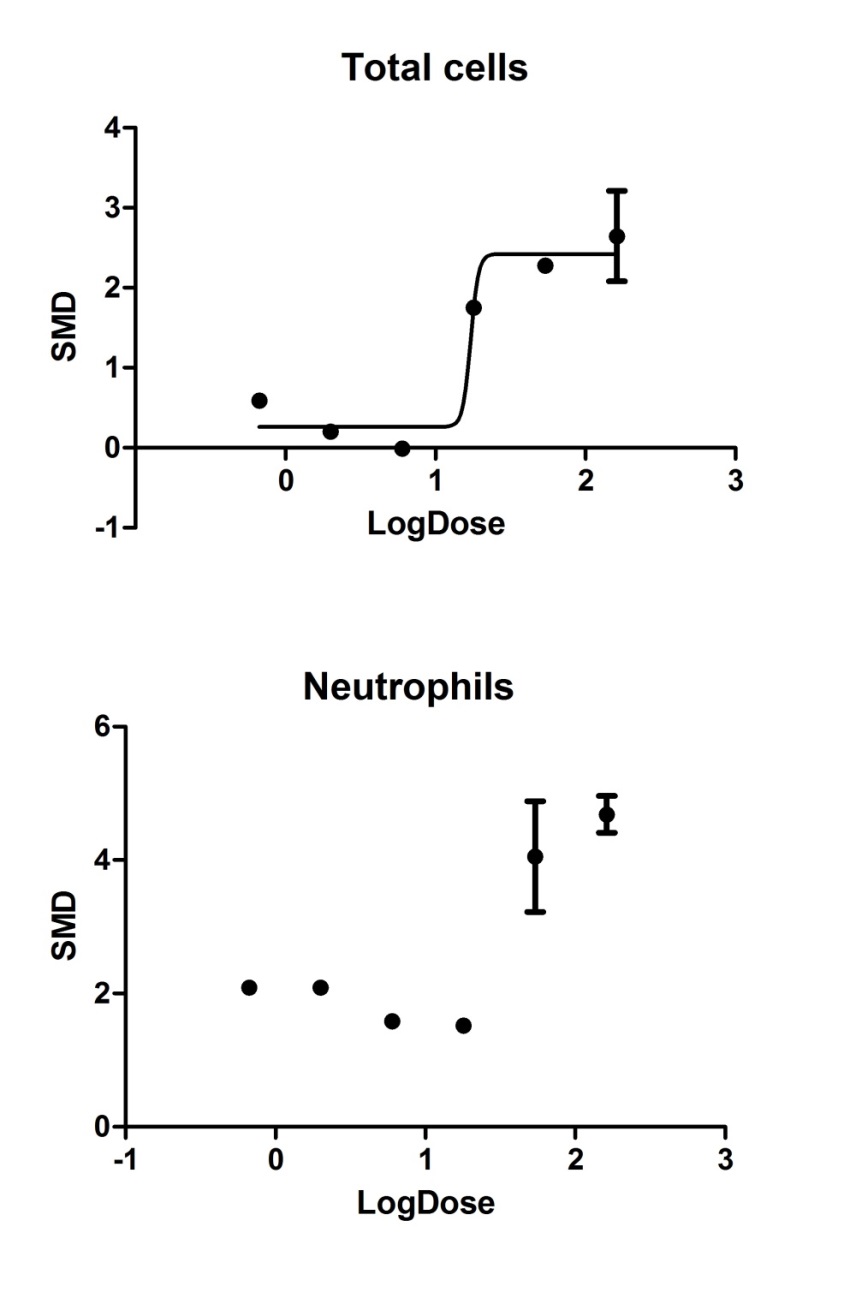

Supplement: S2 Fig — Standardized mean difference (SMD) in total cells (top) or neutrophils (bottom) in BALF as a function of the dose of Printex 90 administered by intratracheal instillation in C57BL/6 mice (0.67, 2.6, 18, 54 or 162 μg/mouse). The animals were sacrificed at 24 h post-exposure. The SMD has been calculated using Review Manager (RevMan) version 5.0 (The Nordic Cochrane Centre. The Cochrane Collaboration. 2008). The SMD is the difference between the two groups divided by the pooled standard deviation. The SMD in the top and bottom graphs cannot be compared with nominal values (i.e. number of cells) because they represent different scales. The SMD for total cells is close to zero (i.e. no effect), whereas there is a slightly increased influx of neutrophils at the low doses. The dose of 18 μg/mouse shows increased total cells as compared to low doses and a similar level of neutrophils. The responses at doses 54 and 162 μg/mouse suggest a plateau for both total cells and neutrophils in BALF. (DOCX) [file pone.0160731.s002.docx]

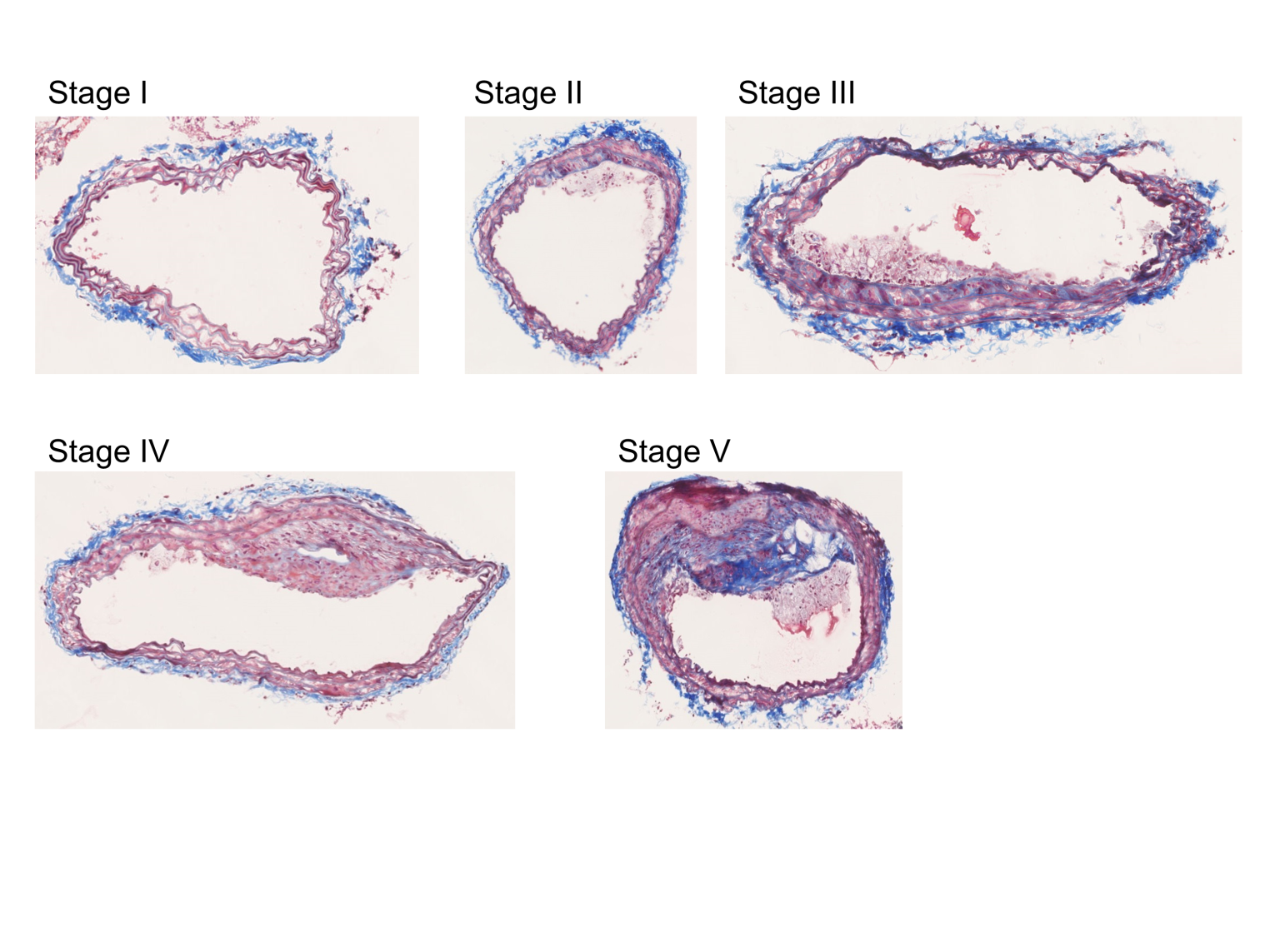

Supplement: S3 Fig — The sections were stained with Masson’s trichrome stain. Classification of atherosclerotic lesion was based on guidelines from American Heart Association. Stages I-III are clinically silent lesions and precursors to advanced lesions. Stage IV is an advanced lesions called atheroma and have a core of accumulated extracellular lipid. Stage V represents advanced lesions called fibroatheroma lesions and has multiple lipid cores, fibrotic layers and calcifications (Stary et al. 1995). (DOCX) [file pone.0160731.s003.docx]
