# Supplementary material for: Inflammation and Vascular Effects after Repeated Intratracheal Instillations of Carbon Black and Lipopolysaccharide
Source: PLoS One. 2016 Aug 29;11(8):e0160731. doi: 10.1371/journal.pone.0160731 (PMC5003393; doi:10.1371/journal.pone.0160731)
Supplement: S1 Table — Upper table shows the BALF cell number and distribution 24 h after a single exposure to LPS. Lower table shows the BALF cell distribution post 24.h after last exposure (one i.t. instillations once a week for 4 weeks). Asterisk denote ***P<0.001. **P<0.01 and *P<0.05 cells influx in exposed group compared to vehicle group. Data are presented as mean ± SEM. Statistical analyses were performed using one-way ANOVA with Tukey’s post-hoc test. (DOCX) [file pone.0160731.s004.docx]

**S1 Table**. **BALF cell number and distribution 24 h post-exposure to LPS.**

| **Dose-response** | **Vehicle** | **0.04 µg** | **0.09 µg** | **0.19 µg** | **0.37 µg** | **0.75 µg** | **1.50 µg** | **3.00 µg** | **6.00 µg** |
| --- | --- | --- | --- | --- | --- | --- | --- | --- | --- |
| Total cells (x 10^3^) | 59.2 ± 2.2 | 179.7 ± 23.7 | 155.0 ± 10.3 | 261.3 ± 30.33 | 298.3 ± 18.2 | 272.7 ± 15.7 | 646.0 ± 110.1*** | 612.0 ± 7.5*** | 453.3 ± 91.3*** |
| Neutrophils (x 10^3^) | 6.8 ± 3.8 | 117.5 ± 21.4 | 97.6 ± 17.4 | 206.3 ± 25.4 | 227.0 ± 22.6 | 220.0 ± 19.0 | 572.3 ± 107.9*** | 558.3 ± 9.9*** | 391.0 ± 103.4*** |
| Macrophage (x 10^3^) | 43.4 ± 4.5 | 45.7 ± 6.2 | 46.2 ± 8.6 | 43.6 ± 8.6 | 52.1 ± 9.0 | 37.0 ± 2.4 | 56.3 ± 8.4 | 36.6 ± 5.0 | 21.8 ±11.4 |
| Eosinophils (x 10^3^) | 0.0 ± 0.0 | 3.4 ± 1.5 | 4.7 ± 3.6 | 3.2 ± 0.8 | 5.8 ± 2.3 | 8.0 ± 2.1 | 8.4 ± 5.2 | 3.1 ± 1.8 | 1.1 ± 1.1 |
| Lymphocytes (x 10^3^) | 0.0 ± 0.0 | 3.3 ± 2.1 | 3.5 ± 2.0 | 0.0 ± 0.0 | 1.5 ± 0.8 | 0.0 ± 0.0 | 0.0 ± 0.0 | 0.0 ± 0.0 | 0.0 ± 0.0 |
| Epithelial (x 10^3^) | 8.8 ± 1.9 | 10.1 ± 4.2 | 5.1 ± 1.2 | 8.2 ± 4.5 | 11.6 ± 4.9 | 7.5 ± 3.3 | 8.6 ± 5.0 | 14.3 ± 7.3 | 9.3 ± 3.4 |
|  |  |  |  |  |  |  |  |  |  |
| **Repeated exposure** | **Vehicle** | **0.80 µg** | **4.00µg** |  |  |  |  |  |  |
| Total cells (x 10^3^) | 273.3 ± 63.9 | 325.7 ± 80.7 | 624.3 ± 56.6* |  |  |  |  |  |  |
| Neutrophils (x 10^3^) | 59.5 ± 27.5 | 220.7 ± 41.7 | 532.7 ± 84.4* |  |  |  |  |  |  |
| Macrophage (x 10^3^) | 80.8 ±18.6 | 75.0 ± 21.1 | 72.0 ± 16.2 |  |  |  |  |  |  |
| Eosinophils (x 10^3^) | 102.3 ± 45.0 | 20.7 ± 15.2 | 16.5 ± 10.0 |  |  |  |  |  |  |
| Lymphocytes (x 10^3^) | 12.2 ± 6.4 | 0.8 ± 0.8 | 0.0 ± 0.0 |  |  |  |  |  |  |
| Epithelial (x 10^3^) | 18.5 ± 9.0 | 8.6 ± 2.1 | 3.5 ± 3.5 |  |  |  |  |  |  |

Upper table shows the BALF cell number and distribution 24 h after a single exposure to LPS. Lower table shows the BALF cell distribution at 24 h after last exposure (one i.t. instillations once a week for 4 weeks). Asterisk denote ***P<0.001, **P<0.01 and *P<0.05 in the exposed group compared to vehicle group. Data are presented as mean ± SEM. Statistical analyses were performed using one-way ANOVA with Tukey’s post-hoc test.
