# Supplementary material for: Inflammation and Vascular Effects after Repeated Intratracheal Instillations of Carbon Black and Lipopolysaccharide
Source: PLoS One. 2016 Aug 29;11(8):e0160731. doi: 10.1371/journal.pone.0160731 (PMC5003393; doi:10.1371/journal.pone.0160731)
Supplement: S2 Table — (DOCX) [file pone.0160731.s005.docx]

**S2 Table**. **Atherosclerosis in the BCA of *ApoE^-/-^* mice at 24 h post-exposure.**

| **Study** | **Vehicle** | **Low-dose CB** | **High-dose CB** | **LPS** | | **Low-dose CB + LPS** | | **High-dose CB + LPS** |  |
| --- | --- | --- | --- | --- | --- | --- | --- | --- | --- |
| **Study 1** |  |  |  |  | |  | |  |  |
| Aorta plaque percentage | 2.8 ± 1.0  (n=10) | 3.1 ± 1.3  (n=10) | 3.3 ± 2.4  (n=10) | 4.6 ± 2.7  (n=10) | | 3.3 ± 2.1  (n=10) | | 1.9 ± 1.1  (n=10) | |
| BCA intima-media ratio | 0.3 ± 0.2  (n=10) | 0.4 ± 0.6  (n=8) | 0.3 ± 0.3  (n=9) | 0.5 ± 0.3  (n=8) | | 0.6 ± 0.3  (n=9) | | 0.6 ± 0.4  (n=9) | |
| BCA intima-lumen ratio | 0.1 ± 0.1  (n=10) | 0.3 ± 0.5  (n=8) | 0.2 ± 0.2  (n=9) | 0.3 ± 0.3  (n=8) | | 0.4 ± 0.3  (n=9) | | 0.3 ± 0.4  (n=9) | |
| BCA plaque percentage in lumen | 9.0 ± 7.3  (n=10) | 15.5 ± 17.6  (n=8) | 15.7 ± 12.5  (n=9) | 19.4 ± 15.0  (n=8) | | 23.3 ± 16.3  (n=9) | | 21.0 ± 17.5  (n=9) |  |
| BCA plaque score | 2.6 ± 1.8  (n=10) | 1.8 ± 1.4  (n=8) | 2.3 ± 1.3  (n=10) | 2.5 ± 1.6  (n=9) | | 2.7 ± 1.6  (n=9) | | 2.2 ± 1.3  (n=9) |  |
| **Study 2** |  |  |  |  |  | | |  |  |
| Aorta plaque percentage | 2.5 ± 1.5  (n=10) | 2.4 ± 1.2  (n=10) |  | 2.5 ± 1.0  (n=9) | |  |  | |  |
| BCA intima-media ratio | 0.5 ± 0.3  (n=8) | 0.3 ± 0.1  (n=6) |  | 0.3 ± 0.1  (n=9) | |  |  | |  |
| BCA intima-lumen ratio | 0.3 ± 0.3  (n=8) | 0.1 ± 0.0  (n=6) |  | 0.1 ± 0.0  (n=9) | |  |  | |  |
| BCA plaque percentage in lumen | 22.3 ± 13.7  (n=8) | 9.7 ± 4.2  (n=6) |  | 9.2 ± 3.8  (n=9) | |  |  | |  |
| BCA plaque score | 2.2 ± 1.4  (n=8) | 1.6 ± 0.9  (n=8) |  | 1.8 ± 0.8  (n=8) | |  |  | |  |
| **Compiled analysis study1 & 2** |  |  |  |  | |  |  | |  |
| Aorta plaque percentage | 2.7 ± 1.3  (n=20) | 2.7 ± 1.3  (n=20) |  | 3.6 ± 2.3  (n=19) | |  |  | |  |
| BCA intima-media ratio | 0.4 ± 0.3  (n=18) | 0.4 ± 0.4  (n=15) |  | 0.4 ± 0.2  (n=17) | |  |  | |  |
| BCA intima-lumen ratio | 0.2 ± 0.2  (n=18) | 0.2 ± 0.3  (n=15) |  | 0.2 ± 0.2  (n=17) | |  |  | |  |
| BCA plaque percentage in lumen | 14.9 ± 12.4  (n=18) | 13.0 ± 13.5  (n=15) |  | 14.0 ± 11.5  (n=17) | |  |  | |  |
| BCA plaque score | 2.4 ± 1.6  (n=18) | 1.7 ± 1.1  (n=18) |  | 2.2 ± 1.3  (n=18) | |  |  | |  |

Data is presented as the mean ± SD and (n) the numbers of animals used in the study. Nested two-way ANOVA was used to analyze the difference between study 1 & 2. P<0.05 was consider as statistical significance. P-values are reported in the result section (not in the table).
